# New Light on an Old Story: Breaking Kasha’s Rule in Phosphorescence Mechanism of Organic Boron Compounds and Molecule Design

**DOI:** 10.3390/ijms23020876

**Published:** 2022-01-14

**Authors:** Dan Deng, Bingbing Suo, Wenli Zou

**Affiliations:** 1 Institute of Modern Physics, Northwest University, Xi’an 710127, China; dengdan@stumail.nwu.edu.cn; 2 Shaanxi Key Laboratory for Theoretical Physics Frontiers, Xi’an 710127, China

**Keywords:** radiative rate constants, non-radiative rate constants, DMRG, MCPDFT, anti-Kasha’s rule

## Abstract

In this work, the phosphorescence mechanism of (E)-3-(((4-nitrophenyl)imino)methyl)-2H-thiochroman-4-olate-BF2 compound (S-BF2) is investigated theoretically. The phosphorescence of S-BF2 has been reassigned to the second triplet state ($T_{2}$) by the density matrix renormalization group (DMRG) method combined with the multi-configurational pair density functional theory (MCPDFT) to approach the limit of theoretical accuracy. The calculated radiative and non-radiative rate constants support the breakdown of Kasha’s rule further. Our conclusion contradicts previous reports that phosphorescence comes from the first triplet state ($T_{1}$). Based on the revised phosphorescence mechanism, we have purposefully designed some novel compounds in theory to enhance the phosphorescence efficiency from $T_{2}$ by replacing substitute groups in S-BF2. Overall, both S-BF2 and newly designed high-efficiency molecules exhibit anti-Kasha $T_{2}$ phosphorescence instead of the conventional $T_{1}$ emission. This work provides a useful guidance for future design of high-efficiency green-emitting phosphors.

## 1. Introduction

Phosphorescence emitters are widely used in organic light-emitting diodes (OLEDs) as photoelectric devices in visualizing, imaging, sensing, and so on due to their 100% internal quantum efficiency (IQE) in theory by fully utilizing triplet excitons of 75% [1,2,3,4]. Traditionally, the most efficient phosphorescence materials are inorganic or organometallic compounds containing heavy atoms like iridium (Ir), platinum (Pt), and gold (Au) [5,6,7,8] which may facilitate intersystem crossing (ISC) between the higher-lying singlet and triplet excited states ($S_{m}$ and $T_{n}$; *m*, *n* = 1, 2, *…*) due to strong spin-orbit coupling (SOC) effects induced by heavy atoms [9]. Unfortunately, these kinds of materials are usually expensive, poisonous, or environmentally unfriendly, and therefore pure organic phosphors may be potential alternatives to substitute organometallic luminophores. Since organic phosphorescence materials are relatively cheap, biocompatible, easy processing, and versatile, they have received extensive attention [10,11]. However, the development of efficient and metal-free room-temperature phosphorescent (RTP) materials is rather challenging [10,12,13,14,15,16,17] because phosphorescent molecules are highly sensitive to temperature and oxygen molecules in the atmosphere. For example, their triplet excited states are extremely unstable at ambient environment and the non-radiative decay and T-T quench may easily take place through thermal collision process and triplet-triplet energy transfer, respectively. Moreover, without the help of heavy atoms, their tiny SOC constants lead to extremely weak ISC from $S_{m}$ to $T_{n}$. Consequently, metal-free RTP materials are rarely applicable.

**Figure 1:**
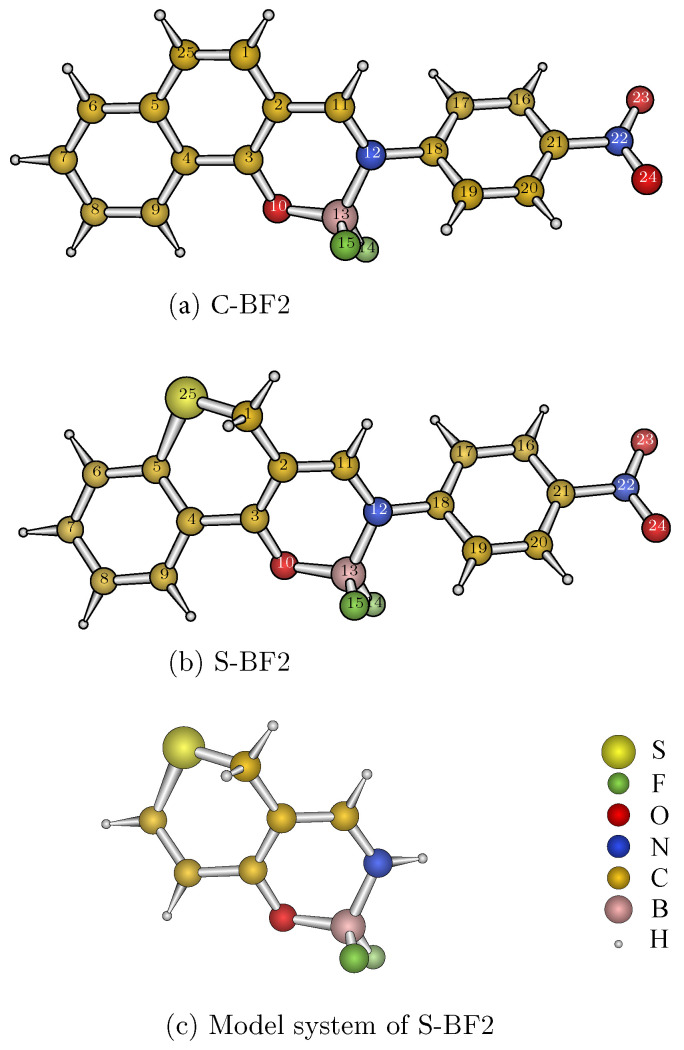
Structures of (**a**) C-BF2, (**b**) S-BF2, and (**c**) the model system.

In recent years, luminescent organoboron compounds have become the focus of investigations because of their promising luminescent properties [2,18,19,20,21,22]. In 2017, Yu et al. [23] synthesized the (E)-3-(((4-nitrophenyl)imino)methyl)-2H-thiochroman-4-olate-BF2 compound (S-BF2; see Figure 1b) based on the parent molecule (E)-2-(((4-nitrophenyl)imino)-methyl)-naphthalen-1-olate-BF2 (C-BF2; Figure 1a), and explored the photoluminescence (PL) mechanism experimentally and theoretically. Although their theoretical proof by time-dependent density functional theory (TDDFT) seems rough, their study opened the door for a new class of laser materials to utilize previously untapped triplet phosphors. In 2018, Paul et al. [24] attributed the luminescence of S-BF2 to the $T_{1}$ state by means of CASSCF(4,4)/NEVPT2 calculations because the widely used TDDFT method, according to them, “cannot”describe multi-reference character in the $S_{1}$ and $T_{1}$ states. Soon afterwards, Lv et al. [25] also revealed the phosphorescence mechanism of S-BF2 from the $T_{1}$ state by TDDFT.

All of the above authors assigned the phosphorescence of S-BF2 to the $T_{1}$ state, but unfortunately, there were some insufficiencies in their studies. In the two TDDFT studies [23,25], for example,

- The vertical emission energy of $T_{1}$ was in poor agreement with the experimental one.
- To improve the agreement of $T_{1}$, Lv et al. tried to use the adiabatic energy rather than the vertical emission one, but only an unusual exchange-correlation (XC) functional could obtain a reasonable result [25].
- Due to Kasha’s rule [26], the probability of $T_{2}$ (as well as higher triplet states) was not considered at all.

Paul et al. contradicted the applicability of TDDFT in S-BF2 [24], but some of their views violates the standard model of quantum chemistry. Before thoroughly exploring the phosphorescence mechanism in S-BF2, here we comment on the work due to Paul et al. [24] and explain the contradiction between CASSCF(4,4)/NEVPT2 and TDDFT.

- Paul et al. claimed that TDDFT fails to get multi-reference $S_{1}$ and $T_{1}$ [24], which is conditional. In principle, a successful TDDFT calculation requires that (1) the reference state (usually $S_{0}$) by density functional theory (DFT) is single-reference characterized, and (2) the interested excited states may be accessed by one or more single-excitations from $S_{0}$. Therefore, an excited state with some multi-reference character may be well calculated by TDDFT only if it is single-excitation dominated from a single-reference $S_{0}$ (for example, see the third reason on page 4518 of Reference [27]), and of course it is also important to choose suitable XC functionals for some systems like transition metal compounds and charge-transfer (CT) excitations.
- CASSCF and NEVPT2 have a different theoretical basis from TDDFT, and both single-reference and multi-reference states may be described in a unified framework. A crucial question in nearly all the multi-configurational methods is whether adequate static correlations can be captured by the active space, and otherwise multi-configurational calculations merely reproduce single-configurational results or even worse. The NEVPT2 emission wavelength of $T_{1}\to S_{0}$ by Paul et al. is 564 nm [24], at first glance being in good agreement with the experimental phosphorescence peak at 575 nm [23], but it is not clear whether all the important π and $\pi^{*}$ orbitals have been included in their quite small active space of (4e,4o).

Due to the above doubts, the phosphorescence mechanism of S-BF2 remains unclear, and it is necessary to explore the emissive mechanism by more accurate approaches.

This paper is structured as follows. In Section 2, we introduce the theoretical methods performed in this study, i.e., TDDFT and high-precision DMRG-SCF/MCPDFT, and provide the fundamental definitions of radiative and non-radiative rate constants. In Section 3, the theoretical results and discussions are grouped into three aspects: (1) the reliability of the cheap TDDFT method is verified with the help of theoretical limit emission energies of S-BF2 by DMRG-SCF/MCPDFT, (2) the PL pathway of S-BF2 is analyzed based on the radiative and non-radiative rates by TDDFT and a revised phosphorescent mechanism is suggested, and (3) a series of new molecules with higher-performance phosphorescence efficiency have been designed theoretically according to the new mechanism. Some conclusions are drawn in Section 4.

## 2. Computational Methods

### 2.1. TDDFT

Geometry optimizations of all the systems (C-BF2, S-BF2 and designed molecules) at the ground and excited states were performed by means of DFT and TDDFT [28], respectively. Vibrational frequencies were also calculated after optimization to ensure that these structures are stable points. To find a suitable XC functional, a series of XC functionals have been examined combined with the basis set 6-311G(d,p) [29,30], including BLYP [31,32], B3LYP [33,34], PBE0 [35,36], CAM-B3LYP [37], ωB97XD [38], recently developed SCAN0 [39,40,41], and so on (see the Supplementary Materials). The experimental spectra of S-BF2 were measured in dichloromethane (CH_2_Cl_2_), so the solvent environment was implicitly simulated by the polarizable continuum model (PCM) [42]. The above DFT and TDDFT calculations were performed using the Gaussian 16 [43] program package, whereas the calculations involving the SCAN0 functional were performed with an in-house BDF program package [44,45,46] (see the Supplementary Materials for the details of SCAN0). The TDDFT/6-311G(d,p) results by different XC functionals may be found in Table S1, and the simulated absorption spectra of S-BF2 have been plotted in Figure S1.

### 2.2. ONIOM(DMRG-SCF/MCPDFT:TDDFT)

To verify the CASSCF(4,4)/NEVPT2 results of Paul et al. [24], we performed the two-layer ONIOM (QM${}_{\mathrm{high}}$:QM${}_{\mathrm{low}}$) combination approach of Morokuma [47,48] as demonstrated in Figure 1b,c, where the geometry of S-BF2 is optimized at the TDDFT(PBE0)/ 6-311G(d,p) level in CH_2_Cl_2_ solvent for $T_{n}$ (*n* = 1 and 2; see Section S6 in the Supplementary Materials). The ONIOM total energy or vertical emission energy of the real system (i.e., S-BF2) in CH_2_Cl_2_ solvent is calculated by

(1) $$E_{\mathrm{real}}^{\mathrm{CH}2\mathrm{Cl}2}\left({\mathrm{QM}}_{\mathrm{High}}:{\mathrm{QM}}_{\mathrm{Low}}\right)\approx E_{\mathrm{model}}^{\mathrm{gas}}\left({\mathrm{QM}}_{\mathrm{High}}\right)-E_{\mathrm{model}}^{\mathrm{gas}}\left({\mathrm{QM}}_{\mathrm{Low}}\right)+E_{\mathrm{real}}^{\mathrm{CH}2\mathrm{Cl}2}\left({\mathrm{QM}}_{\mathrm{Low}}\right)$$

where the low level quantum mechanical method (QM${}_{\mathrm{Low}}$) is TDDFT/6-311G(d,p); two common XC functionals B3LYP and PBE0 are used in this work. In the model system part of calculation by a high level quantum mechanical method (QM${}_{\mathrm{High}}$), the minimal full valence active space consists of 54 electrons in 43 orbitals by neglecting all the core orbitals, deep-lying 2*s* or 3*s* valence orbitals of N, O, F, and S, and high-lying H 1*s* orbitals, i.e., (54e,43o), which is far beyond the capability of CASSCF, and therefore the density matrix renormalization group (DMRG) method is performed instead through self-consistent field orbital optimization iteratively (DMRG-SCF; also loosely called DMRG-CASSCF) with up to 1000 renormalized states (*M* = 1000). The basis set is def2-TZVPP(-f) [49] by eliminating all the *f*-functions from the original one, and the Cholesky decomposition (CD) of the two-electron integrals [50] is used to speed up the calculations with controlled accuracy. Since neither DMRG-NEVPT2 nor DMRG-CASPT2 is feasible for this large active space to capture dynamic correlations, the DMRG based multi-configurational pair-density functional theory (MCPDFT) [51] is carried out subsequently with three different on-top density functionals ftBLYP, ftPBE, and ftrevPBE [52]. It has been found in the literatures that MCPDFT with a suitable active space could perfectly reproduce the CASPT2 results of organic compounds [52,53], d-block transition metal molecules [54,55], and even actinide-containing systems [56]. The OpenMolcas [57,58] program package is used for DMRG-SCF(54e,43o)/MCPDFT calculations by driving the Block program [59,60] as the DMRG solver.

### 2.3. Radiative and Non-Radiative Rates

In order to figure out the radiative and non-radiative photochemical decay processes, the minimal energy crossing points (MECPs) [61] were optimized using the sobMECP procedure [61,62], and the radiative, internal conversion (IC), and ISC rate constants were calculated by the MOMAP program package [63]. A molecule in excited electronic state loses its energy through different relaxation processes, which may be grouped into radiative and non-radiative ones. The radiative process is defined as the emission of photons in a transition between different electronic states, including (spin-conserving) fluorescence and (spin-nonconserving) phosphorescence emissions. On the contrary, there are no emitted photons in the non-radiation process, including (spin-conserving) IC and (spin-nonconserving) ISC [10,64].

The radiative rate (in s${}^{-1}$) is estimated via [10]

(2) $$K_{r}=\frac{3}{2}f{\left(\Delta E\right)}^{2}$$

where the oscillator strength *f* (in atomic unit; a.u.) is defined by

(3) $$f=\frac{2}{3}\mu_{t}^{2}\Delta E$$

In Equations (2) and (3), $\mu_{t}$ is the transition dipole moment in a.u., ΔE is the emission energy in cm${}^{-1}$ (Equation (2)) or a.u. (Equation (3)).

The non-radiative rate is given by the fermi Golden Rule.

(4) $$K_{nr}=2\pi \frac{1}{\hbar }\sum_{f}{\left|H_{if}^{1}\right|}^{2}\delta \left(E_{i}-E_{f}\right)$$

where $H_{if}^{1}$ is the matrix element of the first-order Hamiltonian, being spin-orbit coupling (SOC) matrix element for the ISC rate and non-adiabatic coupling matrix element for the IC rate, the δ function provides the energy conservation for the non-radiative transition, and *i*, *f* represents the initial and final states, respectively.

The SOC constant is an important parameter to calculate $K_{isc}$ and $K_{r}$ in phosphorescent transitions. In this study, the SOC effects were calculated perturbatively by the TDDFT(PBE0) + SOC method implemented in the BDF program package, where the one-center molecular mean-field approximation was applied to the two-electron SO integrals.

## 3. Results and Discussion

### 3.1. Absorption and Emission Energies of S-BF2

In order to verify the previous viewpoint that the phosphorescence of S-BF2 at 575 nm comes from $T_{1}$ [23,24,25], the emission energies and wavelengths of $T_{1}$ and $T_{2}$ are calculated by the DMRG-SCF/MCPDFT based ONIOM combination approach, and the results are summarized in Table 1. It can be seen that the experimental phosphorescence wavelength of S-BF2 at 575 nm [23] may be assigned to the $T_{2}$ state instead of $T_{1}$, which is contrary to all the early results [23,24,25]. This opposing assignment may be attribute to some charge-transfer character [24] and near-degeneracy correlation [65] in the excited states, which may be well described by DMRG/MCPDFT with an adequately large active space [53,66,67].

As for the applicability of DFT/TDDFT to S-BF2, two issues need to be paid attention. It is well known that the accuracy of DFT/TDDFT calculations is generally affected by the amount of multi-reference character in $S_{0}$ and CT character in excited states [68]. The $S_{0}$ state of S-BF2 has single-reference character at its equilibrium geometry [24]. At the $T_{n}$ geometries, however, DMRG-SCF(54e,43o) predicts some multi-reference character in $S_{0}$ of the model system. In addition, the analysis of spatial extent in CT excitations [69] implemented in the Multiwfn program [70] shows that $T_{1}$ and $T_{2}$ are basically local excitations (see Section S3 in the Supplementary Materials). Consequently, DFT/TDDFT may be still applicable for S-BF2 if double-excitations are negligible, but some hybrid XC functionals are preferred to describe both the slight multi-reference character in $S_{0}$ and the modest CT character in the excited states of S-BF2.

Table S1 in the Supplementary Materials collects the emission energies and the corresponding phosphorescence wavelengths of $T_{1}$ and $T_{2}$ at their respective geometries, either in gas phase or in CH_2_Cl_2_ solution. On the whole, the energy of $T_{1}$ state by most of the functionals is too low whereas some common functionals support phosphorescence from the $T_{2}$ state instead of $T_{1}$. Among these functionals, PBE0 exhibits better performance than the others in emission spectra by comparing the wavelengths with the DMRG-SCF(54e,43o)/MCPDFT ones. Therefore, the PBE0 functional will be used in the following study.

At the TDDFT(PBE0) level of theory, the absorption and phosphorescence spectra of S-BF2 are calculated at the $S_{0}$ and $T_{n}$ (*n* = 1 and 2) geometries, respectively, as collected in Table 2. In the absorption spectrum, there are two theoretical peaks at 435 ($S_{0}\to S_{1}$) and 375 nm ($S_{0}\to S_{2}$), which are merely 5 nm blue-shifted compared with the experimental ones at 440 and 380 nm [23] and are better than the CASSCF(4,4)/NEVPT2 results of 410 and 340 nm [24]. Since the oscillator strength (*f*) of 0.632 in the transition $S_{0}\to S_{2}$ is much larger than that of 0.269 in $S_{0}\to S_{1}$, S-BF2 is much easier to be excited to $S_{2}$ than to $S_{1}$. In the phosphorescence spectrum, the experimental peak at 575 nm [23] has been reassigned to the theoretical $T_{2}\to S_{0}$ transition at 559 nm in this study, which has much stronger transition dipole moment (and *f* as well) than that of $T_{1}\to S_{0}$. To confirm the phosphorescence coming from the higher-lying $T_{2}$ state instead of $T_{1}$, radiative and non-radiative rates need to be investigated further in the next subsection.

### 3.2. Radiative and Non-radiative Processes of S-BF2

To deepen the understanding of the PL mechanism in S-BF2, some radiative and non-radiative rate constants are calculated as given in Table 3, including the ISC rate ($K_{isc}$), the IC rate ($K_{ic}$), and the radiative rate ($K_{r}$). In the transition $S_{1}\to S_{0}$, $K_{ic}$ of 5.05 × 10${}^{9}$ s${}^{-1}$ is much faster than $K_{r}$ of 7.37 × 10${}^{7}$ s${}^{-1}$, and therefore the fluorescence probability is relatively weak. In addition, an instantaneous IC process from $S_{2}$ to $S_{1}$ may increase the population on $S_{1}$ further as found in the previous studies [23,24]. On the other hand, $K_{isc}$ of 2.24 × 10${}^{9}$ s${}^{-1}$ in $S_{1}\to T_{2}$ is comparable to $K_{ic}$ in $S_{1}\to S_{0}$ and is far beyond $K_{isc}$ of 1.10 × 10${}^{7}$ s${}^{-1}$ in $S_{1}\to T_{1}$. The $K_{isc}$ rate of $S_{1}\to T_{1}$ is 2.26 × 10${}^{9}$ s${}^{-1}$ in Reference [24], being about 200 times larger than our 1.10 × 10${}^{7}$ s${}^{-1}$ because of the underestimated energy gap between $S_{1}$ and $T_{1}$ by CASSCF/NEVPT2. In our study, the corresponding ISC efficiency ($\Phi_{isc}$) is 47.0% in $S_{1}\to T_{2}$, being about 120 times larger than that of 0.4% in $S_{1}\to T_{1}$ (see Table S12 in the Supplementary Materials), which explains the considerable population on the $T_{2}$ state. Obviously $K_{r}$ = 3.25 × 10${}^{3}$ s${}^{-1}$ surpasses $K_{isc}$ = 1.33 × 10${}^{1}$ s${}^{-1}$ in $T_{2}\to S_{0}$. For comparison, $K_{r}$ = 1.20 × 10${}^{1}$ s${}^{-1}$ in $T_{1}\to S_{0}$ is two orders of magnitude smaller than that in $T_{2}\to S_{0}$, not to mention the agreement of emission wavelength with the experimental one.

According to the Marcus theory, the rate of the internal conversion process $T_{2}\to T_{1}$ is estimated to be about $10^{10}$ s${}^{-1}$, seeming to hinder the $T_{2}\to S_{0}$ radiation processes with the rate of 3.25 × 10${}^{3}$ s${}^{-1}$. However, it is worth noting that the $K_{isc}$($S_{1}\to T_{2}$) rate of 2.24 × 10${}^{9}$ s${}^{-1}$ is so large that the population loss on $T_{2}$ due to $K_{ic}$($T_{2}\to T_{1}$) can be compensated instantaneously. As a consequence, the phosphorescence of the transition $T_{2}\to S_{0}$ may be observed although its radiative rate is much smaller than $K_{ic}$($T_{2}\to T_{1}$). On the other side, the non-radiative rate in $T_{1}$ is much faster than the paltry radiative one with a seriously underestimated emission energy. Compared with $T_{1}\to S_{0}$, $T_{2}\to S_{0}$ has a much larger phosphorescence radiative rate with the better agreement of emission wavelength, and is more suitable for the assignment of the experimental phosphorescence [23]. Consequently, it can be inferred that the phosphorescence process is more likely to come from the $T_{2}$ state according to the above theoretical results, distinctly breaking Kasha’s rule (i.e., fluorescence/phosphorescence usually comes from the lowest excited state [26]).

**Figure 2:**
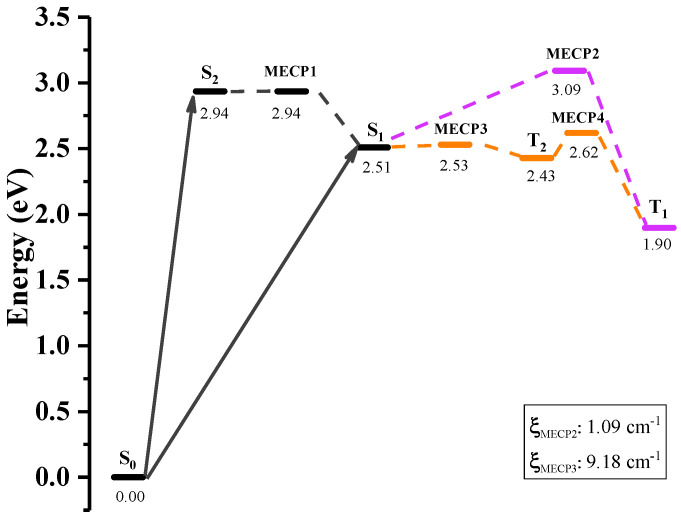
Energies of S-BF2 at the ground and exited states as well as the minimum energy crossing points (MECPs). $\xi_{\mathrm{MECP}}$ represents the SOC constant at MECP.

Although the ISC process is often negligible in the non-radiative transition between the states with different spin multiplicities, it frequently occurs in phosphorescence and may be heavily promoted by remarkable SOC and narrow energy gap [9]. In order to quantitatively unravel the strong non-radiative decay process in $S_{1}\to T_{2}$, the minimum energy crossing points (MECPs) [61] are optimized and the SOC constants at MECPs ($\xi_{\mathrm{MECP}}$) are calculated. The energy profile of non-radiative decay pathway has been plotted in Figure 2. After S-BF2 being excited to the $S_{2}$ state in the Franck-Condon region of $S_{0}$, it relaxes rapidly to the minimum of $S_{2}$ at 2.94 eV, vertically lying only 0.09 eV above $S_{1}$, and then converts to $S_{1}$ by conquering a small barrier at MECP1. From the $S_{1}$ state at 2.51 eV, there are two non-radiative channels to triplet states ($T_{1}$ via MECP2 with a larger barrier of 0.58 eV and $T_{2}$ via MECP3 with a smaller barrier of 0.02 eV), and the corresponding SOC constants at MECPs between $S_{1}$ and $T_{n}$ are 1.09 and 9.18 cm${}^{-1}$, respectively. So the quite large $K_{isc}$ rate in $S_{1}\to T_{2}$ may be attributed to both the low energy barrier and the significant SOC constant at MECP3. For comparison, we have noticed that in C-BF2 the SOC constants between $S_{1}$ and $T_{n}$ are tiny (see Table S8 in the Supplementary Materials), so the ISC process and the subsequent phosphorescence emission are hard to occur. Along with the emission of $T_{2}\to S_{0}$, there is also a fierce competitive processes, i.e., the internal conversion from $T_{2}$ to $T_{1}$ through MECP4.

**Figure 3:**
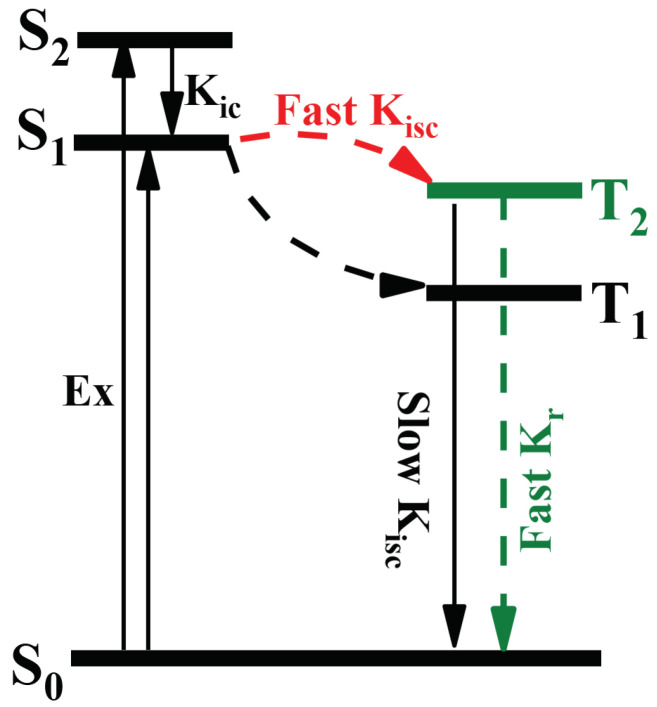
Revised phosphorescence mechanism of S-BF2. $K_{ic}$, $K_{isc}$ and $K_{r}$ refer to internal conversion rate, intersystem crossing rate, and radiative rate, respectively.

In summary, our quantitative results of S-BF2 suggest a revised phosphorescence mechanism $S_{0}\to S_{2}\to S_{1}\to T_{2}\to S_{0}$ as demonstrated in Figure 3. At first, S-BF2 in $S_{0}$ is excited to $S_{1}$ and $S_{2}$ but in the latter state there is an IC process to $S_{1}$. Then an efficient ISC process takes place through the channel $S_{1}\to T_{2}$, and finally anti-Kasha phosphorescence occurs directly in the emission $T_{2}\to S_{0}$. The anti-Kasha phenomenon was found experimentally only in very scarce organic compounds [64,71], e.g., the fluorescence of azulene derivatives [72] and the phosphorescence of N966 [73], ClBDBT [74], and CzCbDBT [75], whereas S-BF2 is a new case according to the above analysis.

### 3.3. Newly Designed S-BF2 Derivatives and Their Photoluminescence Properties

**Figure 4:**
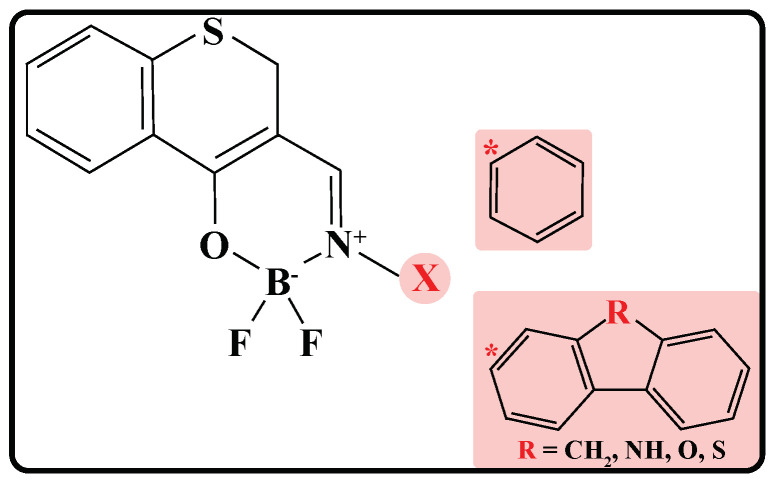
Designed molecules based on S-BF2. The pink part shows the substituents.

The second purpose in this study is to improve S-BF2 theoretically for better phosphorescent performance. The nitrophenyl group in S-BF2 has little effect on the electronic configurations and excitation energies of the S-BF2 frame, and therefore we try to replace the nitrophenyl group with other substituent groups to increase (decrease) the luminous efficiency from $T_{2}$ ($T_{1}$). Since the phenyl, fluorene, carbazole, dibenzofuran, and dibenzothiophene groups are often used as phosphor in experimental synthesis [12,14], they are adopted in this study, leading to S-BF2*, S-BF2_C, S-BF2_N, S-BF2_O, and S-BF2_S, respectively (*cf.* Figure 4). Again, all these molecules are calculated at the TDDFT(PBE0)/6-311G(d,p) level in CH_2_Cl_2_ solution.

The properties of absorption spectra of the designed molecules are listed in Table S11 in the Supplementary Materials. On the whole, these molecules are directly excited to $S_{1}$ because of its larger oscillator strengths, so the IC process from from $S_{2}$ to $S_{1}$ may be ignored. Compared with S-BF2, the $K_{isc}$($S_{1}\to T_{2}$) rates of the new compounds increase dramatically but except S-BF2_S due to larger SOC constants and smaller energy differences, whereas the $K_{isc}$($S_{1}\to T_{1}$) ones decrease by one to three orders of magnitude (see Table S10 in the Supplementary Materials), so the population on $T_{1}$ become irrelevant. At the $T_{2}$ states, the emission energies of the new compounds are about 0.1 eV larger than that of S-BF2, resulting in one to three orders of magnitude reductions in $K_{isc}$($T_{2}\to S_{0}$); the only exception is S-BF2* where $K_{isc}$($T_{2}\to S_{0}$) is five times the S-BF2 one because of larger reorganization energy and stronger SOC constant (see Table S10). At the $T_{1}$ states, in contrast, the $K_{isc}$($T_{1}\to S_{0}$) rates rise considerably with the increases of reorganization energy and reductions of emission energy. On the whole, the $K_{isc}$ rates in $T_{2}\to S_{0}$ are one to seven orders of magnitude weaker than those in $T_{1}\to S_{0}$ so the phosphorescence emission from the latter transition may be difficult to detect. This result may be confirmed further by the radiative rates given in Table 4: the $K_{r}$($T_{1}\to S_{0}$) rates of the new molecules are reduced by several times compared with the S-BF2 one due to the reductions of oscillator strengths, so the parasitic light from $T_{1}\to S_{0}$ can be inhibited effectively. On the contrary, the $K_{r}$ rates in $T_{2}\to S_{0}$ become three to twenty-seven times the S-BF2 one; the only exception is S-BF2* since its oscillator strength of $T_{2}$ is nearly unchanged relative to the S-BF2 one. According to the energy gap law [76], the non-radiative rate of $T_{2}\to T_{1}$ may be significantly reduced as the energy gap increases. Compared with the $K_{isc}$($S_{1}\to T_{2}$) rates with the order of magnitude between nine and ten (see Table S10), the decay of population on $T_{2}$ caused by the much smaller $K_{ic}$($T_{2}\to T_{1}$) rates can be compensated faster (*cf.* Table S9), and therefore the $T_{2}\to S_{0}$ emissions in the new molecules are expected to be detected experimentally. In the phosphorescence spectra summarized in Table 4, the phosphorescence wavelengths from $T_{2}$ of the new molecules are only 1–7 nm red-shifted relative to the one of S-BF2, being still in the range of green light wave band as in S-BF2.

As expected, the PL efficiency of S-BF2 may be significantly improved by replacing the nitrophenyl group with fluorene, carbazole, dibenzofuran, or dibenzothiophene but with a simpler PL process $S_{0}\to S_{1}\to T_{2}\to S_{0}$, where the energy dissipation between $S_{2}$ and $S_{1}$ has been avoided. Experimental syntheses of these new molecules (especially S-BF2_C and S-BF2_N) are highly desirable to verify the phosphorescence mechanism with enhanced PL efficiency.

## 4. Conclusions

In the present paper the PL mechanism of S-BF2 have been systematically and thoroughly investigated. By fully considering the static correlations through DMRG-SCF, dynamic correlations through MCPDFT, and substituents and environments through ONIOM, the previous phosphorescent transition $T_{1}\to S_{0}$ has been reassigned to $T_{2}\to S_{0}$, and the cheaper TDDFT calculations with suitable functionals may also be confirmed. The computed radiative and non-radiative rate constants as well as MECPs suggest a different PL pathway, namely $S_{0}\to S_{2}\to S_{1}\to T_{2}\to S_{0}$, and support the breakdown of Kasha’s rule. Based on the new phosphorescence mechanism, we have purposefully designed some novel compounds to enhance the phosphorescence efficiency from the $T_{2}$ state and reduce the stray light from the $T_{1}$ state by changing the substitutes, which provide a valuable guidance for the design of high-efficiency green-emitting phosphors.

## Figures and Tables

**Table 1:** Emission energies (in eV) and emission wavelengths (in parentheses; nm) of $T_{1}$ and $T_{2}$ of S-BF2 by ONIOM (QM${}_{\mathrm{High}}$:QM${}_{\mathrm{Low}}$).

System	${\mathrm{QM}}_{\mathrm{High}}$ ${}^{\left(a\right)}$	${\mathrm{QM}}_{\mathrm{Low}}$ ${}^{\left(b\right)}$	$T_{1}$	$T_{2}$
Model	A		1.75 (708)	2.40 (516)
	B		1.74 (714)	2.38 (520)
	C		1.77 (702)	2.42 (512)
Real (S-BF2)	A	a	1.87 (664)	2.07 (598)
	A	b	1.93 (643)	2.16 (574)
	B	a	1.85 (669)	2.06 (603)
	B	b	1.92 (647)	2.14 (579)
	C	a	1.88 (659)	2.10 (592)
	C	b	1.94 (638)	2.18 (569)
	Expt. ${}^{\left(c\right)}$		2.16 (575)	

*^(a)^* DMRG-SCF(54e,43o)/MCPDFT with ftBLYP (A), ftPBE (B), or ftrevPBE (C). *^(b)^* TDDFT with B3LYP (a) or PBE0 (b). *^(c)^* Reference [23].

**Table 2:** Excitation energy (in eV), wavelength (in nm), and oscillator strength of S-BF2 in CH_2_Cl_2_ solution.

State	E	λ	*f*	Configuration ${}^{\left(a\right)}$ (%)
Absorption Spectrum				
$S_{1}$	2.85	435	0.269	H→L (93)
Expt. ${}^{\left(b\right)}$	2.82	440		
$S_{2}$	3.30	375	0.632	H-1→L (97)
Expt. ${}^{\left(b\right)}$	3.26	380		
Phosphorescence Spectrum				
$T_{1}$	1.64	758	1.31 × 10${}^{-7}$	H→L (84), H-1→L (11)
$T_{2}$	2.22	559	1.63 × 10${}^{-5}$	H-1→L (53), H→L (38)
Expt. ${}^{\left(b\right)}$	2.16	575		

*^(a)^* H for HOMO and L for LUMO. *^(b)^* Reference [23].

**Table 3:** Radiative and non-radiative rate constants (in s${}^{-1}$) of S-BF2.

Rate	$S_{2}\to S_{1}$	$S_{1}\to S_{0}$	$S_{1}\to T_{1}$	$T_{1}\to S_{0}$	$S_{1}\to T_{2}$	$T_{2}\to S_{0}$
$K_{ic}$	2.80 × 10${}^{13}$	5.05 × 10${}^{9}$				
$K_{isc}$			1.10 × 10${}^{7}$	5.12 × 10${}^{4}$	2.24 × 10${}^{9}$	1.33 × 10${}^{1}$
$K_{r}$		7.37 × 10${}^{7}$		1.20 × 10${}^{1}$		3.25 × 10${}^{3}$

**Table 4:** Emission energies, wavelengths, oscillator strengths, radiative rates, and electron configurations of the new molecules in CH_2_Cl_2_ solution.

Molecule	State	E (eV)	λ (nm)	*f*	$K_{r}$ (s${}^{-1}$)	Configuration ${}^{\left(a\right)}$ (%)
S-BF2*	$T_{1}$	1.44	864	1.65 × 10${}^{-7}$	1.20 × 10${}^{1}$	H→L (95)
	$T_{2}$	2.36	526	2.00 × 10${}^{-5}$	4.40 × 10${}^{3}$	H-1→L (78), H→L(15)
S-BF2_C	$T_{1}$	1.18	1050	6.74 × 10${}^{-8}$	3.42 × 10${}^{0}$	H→L (94)
	$T_{2}$	2.34	530	4.05 × 10${}^{-4}$	8.76 × 10${}^{4}$	H-1→L (89)
S-BF2_N	$T_{1}$	1.21	1025	6.82 × 10${}^{-8}$	3.66 × 10${}^{0}$	H→L (92)
	$T_{2}$	2.35	527	5.47 × 10${}^{-5}$	1.19 × 10${}^{4}$	H-2→L (89)
S-BF2_O	$T_{1}$	1.33	931	7.10 × 10${}^{-8}$	4.57 × 10${}^{0}$	H→L (92)
	$T_{2}$	2.33	533	1.25 × 10${}^{-4}$	2.68 × 10${}^{4}$	H-1→L (90)
S-BF2_S	$T_{1}$	1.35	917	7.44 × 10${}^{-8}$	4.71 × 10${}^{0}$	H→L (92)
	$T_{2}$	2.33	533	8.31 × 10${}^{-5}$	1.77 × 10${}^{4}$	H-2→L (89)

*^(a)^* H for HOMO and L for LUMO.

## Data Availability

The data presented in this study and not reported in the Supplementary Materials are available on request from the corresponding author.

## Supplementary Materials

The following are available at https://www.mdpi.com/article/10.3390/ijms23020876/s1.

## Abbreviations

The following abbreviations are used in this manuscript:

CT	Charge-Transfer
DFT	Density Functional Theory
DMRG	Density Matrix Renormalization Group
IC	Internal Conversion
ISC	Intersystem Crossing
MCPDFT	Multi-Configurational Pair-Density Functional Theory
MECP	Minimum Energy Crossing Point
PL	Photoluminescence
SOC	Spin-Orbit Coupling
TDDFT	Time-Dependent Density Functional Theory
