# Supplementary material for: New Light on an Old Story: Breaking Kasha’s Rule in Phosphorescence Mechanism of Organic Boron Compounds and Molecule Design
Source: Int J Mol Sci. 2022 Jan 14;23(2):876. doi: 10.3390/ijms23020876 (PMC8776103; doi:10.3390/ijms23020876)
Supplement: Supplementary file 1 [file ijms-23-00876-s001.zip › ijms-1516772-supplementary.pdf]

Supplementary Materials

New light on an Old Story: Breaking Kasha's Rule in Phosphorescence Mechanism of Organic Boron Compounds and Molecule Design

Dan Deng,[†,‡] Bingbing Suo,[*,†,‡] and Wenli Zou[*,†,‡]

†*Institute of Modern Physics, Northwest University, Xi'an 710127, Shaanxi, P. R. China*

‡*Shaanxi Key Laboratory for Theoretical Physics Frontiers, Xi'an 710127, Shaanxi, P. R. China*

E-mail: bsuo@nwu.edu.cn; zouwl@nwu.edu.cn

Contents

1 Detail of theoretical calculations **3**

2 Structural parameters of C-BF2 and S-BF2 **7**

3 Charge-transfer indexes of S-BF2 **9**

4 Other results of C-BF2 and S-BF2 **11**

5 Properties of the newly designed molecules **12**

6 Optimized Cartesian coordinates **15**

6.1 C-BF2 15

6.2 S-BF2 19

6.3 S-BF2* 28

6.4 S-BF2_C 33

6.5 S-BF2_N 39

6.6 S-BF2_O 44

6.7 S-BF2_S 50

References **57**

1 Detail of theoretical calculations

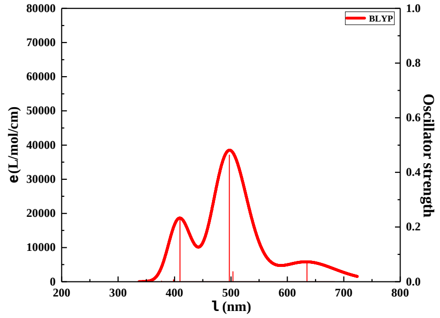

(a) BLYP

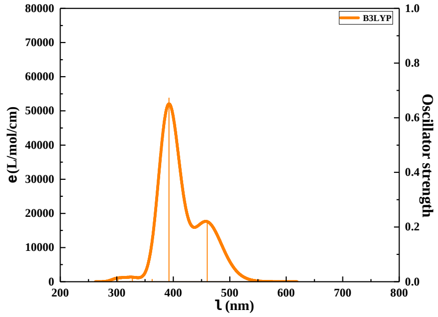

(b) B3LYP

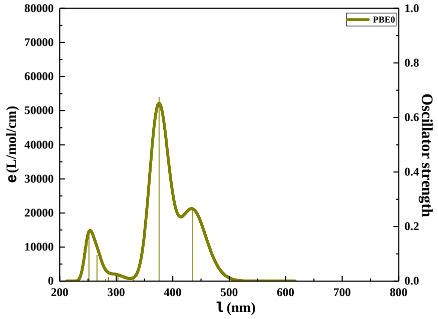

(c) PBE0

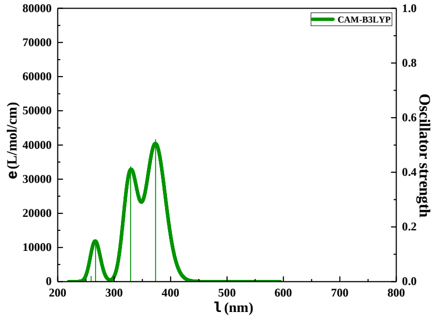

(d) CAM-B3LYP

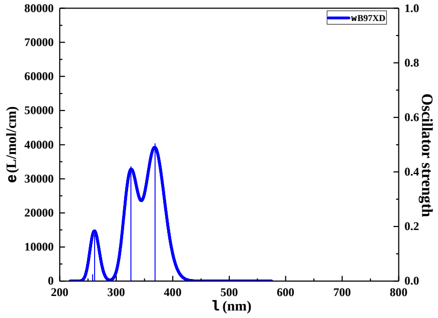

(e) ωB97XD

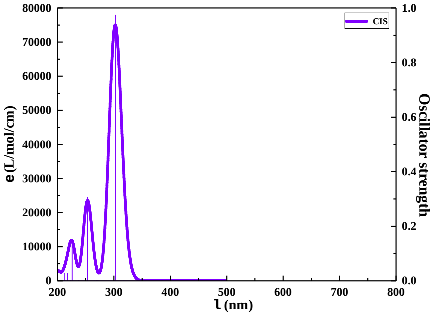

(f) CIS

Figure S1. Absorption spectra of S-BF2 in CH_2Cl_2 solution.

The absorption spectra of S-BF2 by five different XC functionals as well as CIS have been plotted in Figure S1. The excitation energies are excessively underestimated by the pure XC functional BLYP but extremely overestimated by CIS, indicating a positive correlation between the excitation energy and the amount of exact exchange. The absorption spectrum of PBE0 is consistent with the experimental one. A smaller basis set 6-31G(d)[1,2]

Table S1. Emission energies (eV) and wavelengths (nm) of the T_1 and T_2 states of S-BF2 using different XC functionals.

	Gas				CH_2Cl_2			
	**TD-T_1**	**TD-T_2**	**TDA-T_1**	**TDA-T_2**	**TD-T_1**	**TD-T_2**	**TDA-T_1**	**TDA-T_2**
TPSSh	1.52(817)	2.17(571)	1.63(759)	2.21(561)	1.62(766)	2.06(603)	1.70(731)	2.10(589)
B3LYP	1.55(801)	2.27(546)	1.67(742)	2.31(537)	1.66(749)	2.16(575)	1.74(711)	2.20(564)
APFD	1.52(814)	2.30(539)	1.68(738)	2.35(528)	1.64(757)	2.19(566)	1.75(708)	2.24(554)
PBE0	1.52(818)	2.33(533)	1.69(734)	2.38(521)	1.64(758)	2.22(559)	1.77(702)	2.27(546)
SCAN0	1.47(841)[a]	2.36(526)[a]	1.64(754)	2.41(515)	1.61(768)[a]	2.26(548)[a]	1.74(712)	2.31(536)
M06	1.63(762)	2.34(530)	1.76(705)	2.38(520)	1.72(721)	2.22(558)	1.81(684)	2.27(545)
BMK	1.66(746)	2.60(477)	1.83(677)	2.65(467)	1.78(698)	2.50(497)	1.91(648)	2.55(487)
BHandHLYP	1.45(856)	1.84(672)	1.85(669)	2.77(447)	1.61(770)	2.02(614)	1.96(633)	2.68(462)
M06-2X	1.68(739)	2.58(481)	1.85(671)	2.72(455)	1.79(693)	2.57(482)	1.93(643)	2.62(473)
CAM-B3LYP	1.55(802)	2.60(478)	1.81(685)	2.71(458)	1.67(743)	2.53(490)	1.90(654)	2.62(474)
ωB97XD	1.62(763)	2.65(467)	1.86(666)	2.74(452)	1.74(715)	2.58(480)	1.94(639)	2.66(467)
HSE06	1.50(825)	2.28(544)	1.66(745)	2.33(533)	1.62(764)	2.16(573)	1.74(712)	2.21(560)
Expt.						2.16(575)		

[a] The emission energy based on TD-SCAN0 is estimated from the difference between TD-PBE0 and TDA-PBE0.

has also been tested, but the agreements with the experimental values are a little worse (see Table S2). Therefore, the PBE0 functional with the 6-311G(d,p) basis set is the best combination in this testing. All new molecules are also calculated at the TDDFT(PBE0)/6-311G(d,p) level in CH_2Cl_2 solution.

We have also performed some TDDFT and TDDFT-TDA test calculations with different exchange-correlation (XC) functionals as shown in Table S1. On the whole, TDDFT-TDA overestimates the emission energies a little compared with the TDDFT ones, whereas the solvent environment increases T_1 by about 0.1 eV but decreases T_2 by about 0.1 eV in energy. SCAN is the first functional "obeying all 17 known exact constraints"[3] with more physical essence. Hence, T_1 and T_2 results based on SCAN0 agree well with the ONIOM and experimental ones. Unfortunately, the TDDFT(SCAN0) calculation is not available due to the "triplet instability problems" in all the SCAN-based functionals[4] and the third-order derivatives of SCAN in TDDFT-TDA(SCAN0) gradients have not been implemented in BDF. Among these functionals, PBE0 exhibits better performance than the others in both absorption and emission spectra and therefore will be used in the following study. Some calculation details of Gaussian and BDF have been summarized in

Table S3.

Table S2. Absorption wavelengths (λ/nm) and oscillator strengths (f) of singlet excited states of S-BF2 in CH_2Cl_2 solution by two different basis sets.

Excited State	PBE0/6-31G(d,p)		PBE0/6-311G(d,p)		$\Delta\lambda$	Δf	Exp
	λ	f	λ	f			
1	428.9	0.303	435.2	0.269	6.3	-0.0341	440
2	372.8	0.644	375.4	0.675	2.6	0.0306	380
3	330.5	0.003	330.0	0.005	-0.6	0.0025	
4	315.1	0.002	311.0	0.001	-4.1	-0.0004	
5	303.0	0.015	302.8	0.018	-0.3	0.0028	

Table S3. Comparison of calculation details in Gaussian and BDF.

	Gaussian	BDF
DFT Calculation		
Integration Grid	Ultrafine	Fine
Convergence Threshold of Energy	1.0E-8 (tight)	1.0E-8 (tight)
TDDFT Calculation		
Convergence Threshold of Energy	1.0E-4	1.0E-7
Convergence Threshold of Wavefunction	1.0E-2	1.0E-5
Criteria of Optimization		
Maximum Force	0.000450	0.000450
RMS Force	0.000300	0.000300
Maximum Displacement	0.001800	0.001800
RMS Displacement	0.001200	0.001200

The internal conversion rate is computed by the Marcus formula[5–8]

$$K = V^2 \sqrt{\frac{\pi}{\hbar^2 k_B T \lambda}} e^{-\frac{(\lambda+\Delta G)^2}{4k_B T \lambda}} \tag{1}$$

where $\hbar$, κ_B, T, and ΔG represent the Planck constant, Boltzmann constant, temperature and free energy difference, respectively. λ is reorganization energy. Here V is the electronic coupling integral between two states, which may be estimated by means of the

two-state Generialzed Mulliken-Hush (GMH) approach[9,10]

$$V = \frac{\mu_t \Delta E}{\sqrt{\Delta\mu^2 + 4\mu_t^2}} \tag{2}$$

where $\Delta\mu$ and ΔE are the dipole moment difference and energy difference between the two states, respectively.

2 Structural parameters of C-BF2 and S-BF2

Some important geometric parameters of C-BF2 and S-BF2 have been collected in Table S4. It can be seen that the geometric parameters in gas phase and solvent environment are almost the same, indicating little influence of solvent effect on their structures no matter in ground or excited states. Since the experimental spectra were measured in CH_2Cl_2 solvent, only the results in CH_2Cl_2 solvent will be discussed. For the both molecules, significant differences exist in the bond lengths, bond angles, and dihedral angles involving C25 and S25 (see Figure 1 in the text about atomic numbering), while the differences in the other parameters are negligible.

Table S4. Bond lengths (in Å), bond angles, and dihedral angles (in degree) of the ground and excited states of C-BF2 and S-BF2 in different environments.

**Mol.**	**Parameter**	**Gas**				**CH_2Cl_2**			
		S_0	S_1	T_1	T_2	S_0	S_1	T_1	T_2
C-BF2	C5-C25	1.430	1.396	1.421	1.400	1.429	1.404	1.421	1.401
	C25-C1	1.356	1.407	1.377	1.384	1.357	1.398	1.379	1.377
	N12-C18	1.415	1.393	1.382	1.354	1.417	1.388	1.384	1.356
	C21-N22	1.469	1.445	1.459	1.450	1.464	1.430	1.449	1.434
	C5-C25-C1	120.4	120.3	122.0	120.3	120.8	120.7	122.0	120.3
	C6-C5-C25-C1	-179.6	-179.7	-179.8	-179.1	-179.5	-179.7	-179.8	-179.3
	C11-N12-C18-C19	140.3	153.3	157.1	160.2	139.7	156.3	156.6	159.5
	C16-C21-N22-O24	-179.4	-178.5	-178.7	-176.7	-179.0	-178.2	-178.2	-175.4
S-BF2	C5-S25	1.761	1.735	1.763	1.740	1.761	1.727	1.748	1.719
	S25-C1	1.816	1.795	1.812	1.847	1.816	1.791	1.805	1.817
	N12-C18	1.410	1.389	1.362	1.398	1.411	1.384	1.383	1.385
	C21-N22	1.467	1.454	1.466	1.457	1.461	1.441	1.457	1.446
	C5-S25-C1	97.9	104.1	98.3	100.8	98.1	104.2	100.9	100.7
	C6-C5-S25-C1	154.5	163.4	152.0	154.4	154.5	166.2	158.6	154.7
	C11-N12-C18-C19	141.9	150.9	158.8	149.8	142.9	154.0	153.7	152.7
	C16-C21-N22-O24	-179.3	-179.3	-178.6	-179.0	-179.3	-178.2	-178.0	-178.5

For C-BF2, the structural relaxation is very small from S_1 to T_n (n=1,2). Compared to the geometry at S_0, the changes in bond lengths and bond angles are smaller than 0.05 Å and 1.7°, respectively, while the maximum deviations of dihedral angle take place in D(C11-N12-C18-C19), being about 17°, which is closely connected with the fairly large

non-radiative decay rate $K_{ic}(S_1 \rightarrow S_0)$ (see Table S5).

Table S5. Radiative and non-radiative rates (in s^{-1}) of C-BF2.

Rate	$S_1 \rightarrow S_0$	$S_1 \rightarrow T_1$	$T_1 \rightarrow S_0$	$S_1 \rightarrow T_2$	$T_2 \rightarrow S_0$
K_{ic}	3.64×10^9				
K_{isc}		1.15×10^4	1.07×10^3	7.87×10^7	6.76
K_r	1.03×10^8		8.20×10^{-1}		4.96×10^1

For S-BF2, the change of D(C6-C5-S25-C1) is 7.6° during the relaxation from S_1 to T_1 but 11.6° from S_1 to T_2, whereas the changes in the other key structural parameters are relatively small. Thus D(C6-C5-S25-C1) should be the dominant factor of structural relaxation from S_1 and T_n (n=1,2), meaning that the S atom plays a key role in the ISC process. The geometry deformations of D(C11-N12-C18-C19) mainly lead to non-radiative processes $T_n \rightarrow S_0$, being 10.8° for T_1 and 9.8° for T_2, respectively. As for D(C6-C5-S25-C1), it is almost unchanged in T_2 relative to in S_0 and becomes 4.1° larger in T_1. These structural distortions between the T_n and S_0 states are positively related to the stokes shifts in absorption and emission spectra of S-BF2.

3 Charge-transfer indexes of S-BF2

For S-BF2, the analysis of CT excitation based on density difference was performed at the DFT/TDDFT(PBE0) level of theory using the MULTIWFN program.[11] The CT indexes are summarized in Table S6, including D_{CT} (distance between the barycenters of ρ_+ and ρ_-), q_{CT} (transferred charge), t_{CT} (separation degree of $\rho_+ = q_{CT}$ and $\rho_- = -q_{CT}$), and $S_\pm$ (overlap of C_+ and C_- functions). The results of the model system (see Figure 1 in the text) are also listed for comparison, where the DMRG-SCF natural orbitals were used to compute density difference between S_0 and T_n.

Table S6. CT indexes for S-BF2 in CH_2Cl_2 solution and the model system.

System	State	D_{CT} (Å)	$\|q_{CT}\|$ (e)	t_{CT} (Å)	$S_\pm$
Model system in gas	T_1	0.432	0.637	-0.804	0.978
	T_2	1.419	0.754	-0.309	0.829
S-BF2 in CH_2Cl_2	T_1	1.094	0.586	-1.383	0.958
	T_2	2.409	0.726	-0.312	0.843

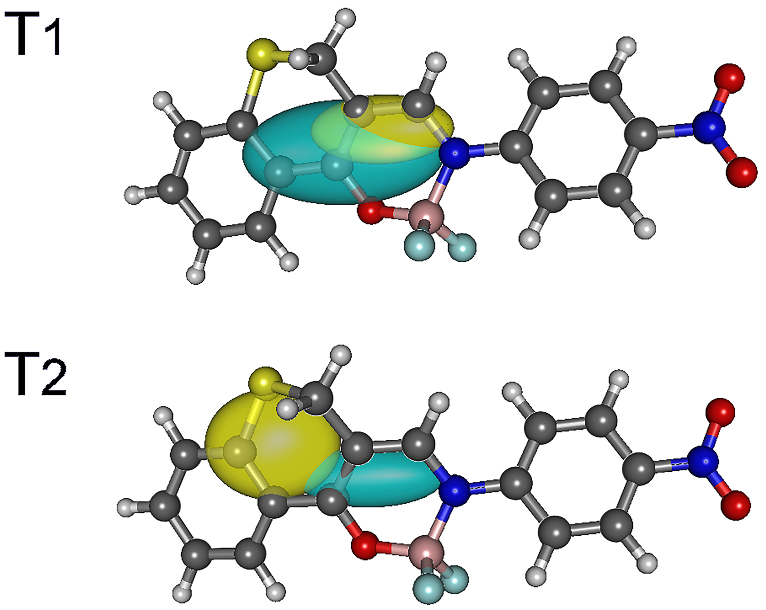

Figure S2. Isosurfaces of C_+ (blue) and C_- (yellow) functions with the isocontour value of 0.0013 a.u.

The D_{CT} and q_{CT} indexes of S-BF2 are not very large, showing that T_1 and T_2 are short-range excitations where the CT character does not make important contributions. Negative t_{CT} indexes with $S_{\pm} \approx 1$ indicate that the barycenters of ρ_+ and ρ_- are not substantially separated (see Figure S2). Since the $|t_{CT}|$ indexes are much smaller than the reference upper-limit of about 1.6 Å (*cf.* J. Chem. Theory Comput. 7, 2498, 2011), DFT/TDDFT should be reasonable to describe T_1 and T_2 of S-BF2.

4 Other results of C-BF2 and S-BF2

At the TDDFT(PBE0)/6-311G(d,p) level of theory, the absorption and phosphorescence spectra of C-BF2 in CH_2Cl_2 solution are summarized in Table S7, and the SOC constants C-BF2 and S-BF2 are given in Table S8. Results of the dark state S_2 are not provided.

Table S7. Absorption and emission energies (eV), wavelengths (nm), oscillator strengths, and electron configurations of C-BF2 in CH_2Cl_2 solution.

State	ΔE	λ	f	Configuration (%)
Absorption Spectrum				
S_1	2.91	426	0.331	H→L (97)
Expt.	2.88	430		
S_3	3.82	325	0.355	H-1→L (84), H→L+1 (9)
Expt.	3.71	334		
Emission Spectrum				
S_1	2.41	515	0.456	H→L (98)
Expt.	2.41	515		

Table S8. SOC constants (cm^{-1}) of C-BF2 and S-BF2 in CH_2Cl_2 solution.

ξ	C-BF2	S-BF2
$<S_0\|H_{SO}\|T_1>$	2.06	7.67
$<S_0\|H_{SO}\|T_2>$	0.41	13.37
$<S_1\|H_{SO}\|T_1>$	0.29	6.35
$<S_1\|H_{SO}\|T_2>$	1.02	9.29

5 Properties of the newly designed molecules

Table S9. The electronic coupling (in eV), free energy difference (in cm^{-1}), reorganization energy (in eV), and internal conversion rate constants (s^{-1}) from $T_2 \rightarrow T_1$ of these molecules.

**Molecule**	**V**	ΔG	ϵ	K_{ic}
S-BF2	0.17	0.5961	0.1773	8.15×10^{10}
S-BF2*	0.33	0.8000	0.2111	5.11×10^{8}
S-BF2_C	0.44	0.9203	0.2330	2.11×10^{7}
S-BF2_N	0.44	0.9150	0.2358	4.16×10^{7}
S-BF2_O	0.39	0.8357	0.2114	1.02×10^{8}
S-BF2_S	0.39	0.8265	0.2061	8.24×10^{7}

For the newly designed molecules as well as S-BF2, some properties in the transitions $S_1 \rightarrow T_n$ and $T_n \rightarrow S_0$ (n=1,2) are summarized in Table S10, and the results of absorption spectra may be found in Table S11.

The intersystem crossing quantum efficiency from S_1 to T_n may be estimated by the formula[12]

$$\Phi_{isc}(S_1 \rightarrow T_2) = \frac{K_{isc}(S_1 \rightarrow T_2)}{K_{isc}(S_1 \rightarrow T_2) + K_r(S_1 \rightarrow S_0) + K_{ic}(S_1 \rightarrow S_0) + K_{isc}(S_1 \rightarrow T_1)} \quad (3)$$

as given in Table S12.

Table S10. Intersystem crossing rates (s^{-1}), adiabatic energy differences (eV), SOC constants (cm^{-1}), and reorganization energies (cm^{-1}) of S-BF2 and the new molecules.

Transition	Molecule	K_{isc}	ΔE	ξ	ϵ
$S_1 \rightarrow T_1$	S-BF2	1.11×10^{7}	0.613	6.353	1022
	S-BF2*	3.85×10^{6}	0.841	4.769	2060
	S-BF2_C	4.90×10^{4}	0.823	2.167	1085
	S-BF2_N	9.00×10^{4}	0.809	2.444	1026
	S-BF2_O	8.90×10^{4}	0.799	2.292	961
	S-BF2_S	1.72×10^{5}	0.799	2.490	1004
$S_1 \rightarrow T_2$	S-BF2	2.24×10^{9}	0.081	9.294	4364
	S-BF2*	2.06×10^{10}	0.089	10.178	2820
	S-BF2_C	2.54×10^{10}	0.062	7.044	2512
	S-BF2_N	2.97×10^{9}	0.074	2.282	2673
	S-BF2_O	1.65×10^{10}	0.006	8.244	2150
	S-BF2_S	1.92×10^{9}	0.015	2.789	2008
$T_1 \rightarrow S_0$	S-BF2	5.12×10^{4}	1.897	7.672	4348
	S-BF2*	1.29×10^{6}	1.816	4.557	5938
	S-BF2_C	1.62×10^{7}	1.660	3.505	7210
	S-BF2_N	1.49×10^{7}	1.675	3.922	7023
	S-BF2_O	3.54×10^{6}	1.741	4.110	6343
	S-BF2_S	5.34×10^{6}	1.751	3.966	6366
$T_2 \rightarrow S_0$	S-BF2	3.23×10^{2}	2.429	13.369	3448
	S-BF2*	1.67×10^{3}	2.568	21.648	3481
	S-BF2_C	5.74×10^{-1}	2.545	17.071	3401
	S-BF2_N	3.41×10^{-2}	2.558	2.995	3455
	S-BF2_O	9.57×10^{-1}	2.535	16.952	3490
	S-BF2_S	1.58×10^{-2}	2.535	1.894	3434

Table S11. Absorption energies (eV), wavelengths (nm), oscillator strengths and electron configurations of S-BF2 and the new molecules.

Molecule	State	ΔE	λ	f	Configuration (%)
S-BF2	S_1	2.85	435	0.269	H → L (93)
	S_2	3.30	375	0.632	H-1→L (97)
S-BF2*	S_1	3.03	410	0.252	H → L (93)
	S_2	3.45	360	0.441	H-1→L (93)
S-BF2_C	S_1	2.91	426	0.675	H→L (85), H-1→L (14)
	S_2	3.21	386	0.163	H-1→L (84), H→L (14)
S-BF2_N	S_1	2.91	426	0.673	H→L (52), H-1→L (33), H-2→L (13)
	S_2	3.03	409	0.011	H-1→L (55), H→L (42)
S-BF2_O	S_1	2.94	421	0.564	H→L (81), H-1→L (17)
	S_2	3.25	382	0.334	H-1→L (80), H→L (18)
S-BF2_S	S_1	2.95	421	0.547	H→L (80), H-2→L (17)
	S_2	3.24	382	0.208	H-2→L (48), H-1→L (33), H→L (17)

Table S12. Intersystem crossing quantum efficiency from S_1 to T_n (n=1,2).

Molecule	$\Phi_{isc}(S_1 \rightarrow T_1)$	$\Phi_{isc}(S_1 \rightarrow T_2)$
C-BF2	3.01×10^{-6}	0.0216
S-BF2	0.0040	0.4697
S-BF2*	0.0002	0.8292
S-BF2_C	3.09×10^{-6}	0.8780
S-BF2_N	2.06×10^{-5}	0.4488
S-BF2_O	7.37×10^{-6}	0.8180
S-BF2_S	4.48×10^{-5}	0.3299

6 Optimized Cartesian coordinates

6.1 C-BF2

Table S13. The S_0 state Cartesian coordinates of C-BF2 in CH_2Cl_2.

	X	Y	Z
C	-2.35851500	2.26852000	0.56401300
C	-1.61361600	1.07697000	0.31467900
C	-2.27623800	-0.11589100	-0.01746500
C	-3.69941000	-0.12835000	-0.12528700
C	-4.41507400	1.07293800	0.13027400
C	-5.82041100	1.04622900	0.02703000
H	-6.37717500	1.95728600	0.22086600
C	-6.47955600	-0.11241400	-0.31437900
C	-5.76427400	-1.29724900	-0.56600400
C	-4.39370200	-1.30579400	-0.47139800
H	-7.56184400	-0.11401900	-0.38954900
H	-6.29723800	-2.20304000	-0.83266000
H	-3.82610900	-2.20944500	-0.65868400
H	-1.81780400	3.17379400	0.81991200
O	-1.61716800	-1.21836500	-0.26568500
C	-0.20567200	1.07909100	0.32062700
N	0.50777500	-0.00414700	0.14140800
B	-0.19756800	-1.41687700	0.04758500
F	0.39199100	-2.15025200	-0.95919200
F	-0.07318100	-2.05098200	1.27909200
C	3.90459100	1.20162800	-0.71431700
C	2.52441900	1.11807600	-0.65167600
C	1.92010100	0.07926600	0.05814300
C	2.69876900	-0.89709100	0.68287800
C	4.07781700	-0.82050900	0.61712900
C	4.66132100	0.23085700	-0.07616100
H	4.39533100	1.99459500	-1.26277800
H	1.91892800	1.84349300	-1.18256800
H	2.21757600	-1.69606200	1.23164800
H	4.70334500	-1.55864600	1.10125100
N	6.12097500	0.31303100	-0.14423900
O	6.60809100	1.24996800	-0.74629000

O	6.76366800	-0.55924300	0.40615600
C	-3.71270500	2.26669300	0.48065500
H	-4.28123000	3.17008500	0.67153200
H	0.31972500	2.01855500	0.47792800

Table S14. The S_1 state Cartesian coordinates of C-BF2 in CH_2Cl_2.

	X	Y	Z
C	-2.37116700	2.26978900	0.40689300
C	-1.61592000	1.11135900	0.23199200
C	-2.29417700	-0.11107300	-0.02027600
C	-3.71796400	-0.14667000	-0.10948200
C	-4.45925400	1.06103300	0.07076000
C	-5.86850500	1.02370500	-0.01500000
H	-6.42461300	1.94461500	0.12511600
C	-6.53032700	-0.16309700	-0.27277600
C	-5.79692000	-1.34102700	-0.44819700
C	-4.40766900	-1.33641500	-0.36698500
H	-7.61165800	-0.18112000	-0.33746800
H	-6.31373200	-2.27264200	-0.64927200
H	-3.84719900	-2.25369200	-0.50233200
H	-1.86186300	3.20789100	0.60064200
O	-1.62287200	-1.21080900	-0.19360300
C	-0.19167300	1.14404200	0.27071200
N	0.54030700	0.04018900	0.13064700
B	-0.17659400	-1.36961100	0.09615700
F	0.36130400	-2.13881400	-0.91141200
F	-0.04436900	-1.96960900	1.33948300
C	3.95291200	1.33127400	-0.44538300
C	2.58011400	1.27573500	-0.38693700
C	1.92384600	0.10000700	0.03974900
C	2.70335000	-1.02427800	0.38863200
C	4.07599200	-0.96553600	0.33630700
C	4.70586700	0.21392900	-0.07490700
H	4.46156700	2.22096400	-0.79251700
H	2.00764500	2.13185400	-0.72250000
H	2.21926600	-1.92655800	0.73541300
H	4.68055400	-1.81689200	0.61942800
N	6.13342500	0.27511500	-0.12886300
O	6.66129700	1.33194000	-0.47323600
O	6.77277100	-0.73199700	0.17077700

C	-3.76703200	2.25448500	0.33340200
H	-4.32075300	3.17545600	0.47587300
H	0.30679700	2.09021400	0.43916900

Table S15. The T_1 state Cartesian coordinates of C-BF2 in CH_2Cl_2.

	X	Y	Z
C	-2.36489300	2.28805400	0.39683300
C	-1.60027600	1.12408600	0.21792300
C	-2.29986900	-0.11622900	-0.03046600
C	-3.71981900	-0.15615700	-0.11225600
C	-4.45271800	1.05096700	0.06799300
C	-5.85037600	1.03096000	-0.00522400
H	-6.39739900	1.95759900	0.13618100
C	-6.53217700	-0.15623500	-0.25463000
C	-5.81250400	-1.33564100	-0.43054900
C	-4.42173000	-1.34458100	-0.36102400
H	-7.61420700	-0.16129300	-0.31020000
H	-6.33936600	-2.26368100	-0.62453800
H	-3.86944800	-2.26631800	-0.49788600
H	-1.85773200	3.22726900	0.58854200
O	-1.61864700	-1.21930800	-0.20660500
C	-0.19968100	1.15790100	0.25837600
N	0.53912100	0.03401500	0.12178800
B	-0.17969800	-1.37212600	0.08866600
F	0.37105000	-2.14295000	-0.91537600
F	-0.04230100	-1.97454500	1.33347800
C	3.95027700	1.33205300	-0.43766600
C	2.57480200	1.27647100	-0.38045600
C	1.91974300	0.10077300	0.04341000
C	2.69783300	-1.02339000	0.39365400
C	4.07232300	-0.96511400	0.34055100
C	4.69306600	0.21291800	-0.07097800
H	4.45963900	2.22377900	-0.77846600
H	2.00221000	2.13357400	-0.71118500
H	2.21138100	-1.92461500	0.73906200
H	4.67712000	-1.81656500	0.62321000
N	6.13983800	0.27320700	-0.12625400
O	6.65738700	1.32557500	-0.46349600
O	6.76677700	-0.73100800	0.16775900
C	-3.74177400	2.25447200	0.32539300

H	-4.30545900	3.17111200	0.46553800
H	0.30281000	2.09674000	0.44818600

Table S16. The T_2 state Cartesian coordinates of C-BF2 in CH_2Cl_2.

	X	Y	Z
C	-2.38497600	2.28203700	0.45157300
C	-1.60442100	1.09797300	0.25075500
C	-2.26811800	-0.10685400	-0.03533600
C	-3.67856400	-0.14074500	-0.14822300
C	-4.43509500	1.07271300	0.05294000
C	-5.85115600	1.02282600	-0.06347100
H	-6.41452100	1.93719700	0.09076400
C	-6.48812200	-0.14841600	-0.36179900
C	-5.73537800	-1.33677000	-0.55785400
C	-4.36515500	-1.33357700	-0.45135800
H	-7.56798900	-0.17918000	-0.44977600
H	-6.25316600	-2.26003700	-0.79412000
H	-3.79386500	-2.24195900	-0.59769400
H	-1.86770800	3.20946600	0.67505900
O	-1.58371200	-1.21678600	-0.23768700
C	-0.21000600	1.13778000	0.29155800
N	0.55095500	0.00599600	0.15674700
B	-0.20326500	-1.39638700	0.20343100
F	0.40322900	-2.28127400	-0.66937700
F	-0.15577100	-1.86146500	1.51221700
C	3.91922700	1.35825500	-0.42984200
C	2.55728500	1.29623700	-0.34721300
C	1.89981300	0.08310500	0.04265300
C	2.71140400	-1.06075400	0.34040300
C	4.07206100	-0.98722500	0.26813400
C	4.68450000	0.22147100	-0.11520700
H	4.42249700	2.26186500	-0.74678100
H	1.97517400	2.16304400	-0.63061500
H	2.23584900	-1.97545300	0.66241300
H	4.69329900	-1.83772400	0.51451900
N	6.11471600	0.29618000	-0.18802900
O	6.62240500	1.38245500	-0.44297400
O	6.75448800	-0.73044300	0.00685800
C	-3.75893600	2.26545100	0.35940800
H	-4.32611600	3.17623500	0.51452100

H	0.29443700	2.07504800	0.48567200

6.2 S-BF2

Table S17. The S_0 state Cartesian coordinates of S-BF2 in CH_2Cl_2.

	X	Y	Z
C	-2.22903200	2.00184700	1.12402000
C	-1.42734100	0.84971600	0.61030300
C	-2.06428500	-0.30328700	0.15779700
C	-3.50542000	-0.36738000	-0.04289800
C	-4.28585400	0.80333100	-0.15224000
C	-5.63948800	0.69429800	-0.46905700
H	-6.24003000	1.59290100	-0.55849300
C	-6.22069900	-0.55151400	-0.64953600
C	-5.46284200	-1.71389000	-0.51545000
C	-4.11608400	-1.61687300	-0.22235700
H	-7.27793800	-0.61554400	-0.88358500
H	-5.92388500	-2.68637100	-0.64279000
H	-3.50457500	-2.50630800	-0.12866300
H	-1.61938600	2.90330800	1.20074200
O	-1.39276200	-1.36135800	-0.17843500
C	-0.03810700	0.89456200	0.56097200
N	0.71372800	-0.14869800	0.25079400
B	0.05051900	-1.55759800	0.10216000
F	0.60971500	-2.23812700	-0.95700900
F	0.18564800	-2.26515200	1.29037400
C	4.02213900	1.28422200	-0.59877200
C	2.65064700	1.14594600	-0.47918400
C	2.10967800	-0.00219600	0.10537100
C	2.95191600	-1.02800500	0.54504300
C	4.32190800	-0.89773900	0.41924300
C	4.84026900	0.25968000	-0.14643100
H	4.46021800	2.16272300	-1.05364200
H	2.00030400	1.91905900	-0.87147700
H	2.52589300	-1.91087000	1.00380600
H	4.99195800	-1.67505800	0.76192600
N	6.28891100	0.39931900	-0.27717200
O	6.71850600	1.42520900	-0.76930000
O	6.98525500	-0.51719500	0.11473100
S	-3.59955700	2.41709000	0.00780900

H	0.46966200	1.82755200	0.79393700
H	-2.65105400	1.78877700	2.10994100

Table S18. The S_1 state Cartesian coordinates of S-BF2 in CH_2Cl_2.

	X	Y	Z
C	-2.13194300	2.19284400	0.65732200
C	-1.40484600	0.95183300	0.28896300
C	-2.05923200	-0.24754700	-0.00621800
C	-3.48848100	-0.38222900	-0.11690700
C	-4.37884200	0.73152500	-0.06386300
C	-5.76051900	0.56566900	-0.20482900
H	-6.40792800	1.43484200	-0.15637700
C	-6.29364800	-0.69742800	-0.39985300
C	-5.43520900	-1.79883100	-0.45395300
C	-4.06188200	-1.64339700	-0.31463800
H	-7.36407500	-0.82503500	-0.50532200
H	-5.84315300	-2.79175400	-0.60754600
H	-3.40447200	-2.50182200	-0.36174600
H	-1.61323200	3.08463200	0.29453700
O	-1.35608200	-1.34623000	-0.23254100
C	-0.01470300	1.00534400	0.32514800
N	0.75233900	-0.08691000	0.14971200
B	0.04931000	-1.49754200	0.14908200
F	0.65388800	-2.32649500	-0.78130800
F	0.14295700	-2.03974700	1.43236500
C	4.10750500	1.31661400	-0.49065000
C	2.73592700	1.21266400	-0.41640800
C	2.12701600	0.02493600	0.04056300
C	2.94978100	-1.06312200	0.40139400
C	4.31985400	-0.95794600	0.32865500
C	4.89781500	0.23328000	-0.11231700
H	4.58024600	2.21867600	-0.85654100
H	2.12784800	2.04081600	-0.75960700
H	2.49748900	-1.97356900	0.76936600
H	4.95764900	-1.78198200	0.62044000
N	6.33280000	0.34301700	-0.18688000
O	6.81389800	1.40753200	-0.55261500
O	7.00388100	-0.63319700	0.11944700
S	-3.82342400	2.35973000	0.09262900
H	0.47820500	1.94832100	0.53363500

H	-2.19305600	2.29224500	1.75356300

Table S19. The S_2 state Cartesian coordinates of S-BF2 in CH_2Cl_2.

	X	Y	Z
C	-2.25840600	2.01147500	1.09733700
C	-1.43856500	0.86676400	0.57116500
C	-2.10940200	-0.31646900	0.13091600
C	-3.54014400	-0.37956300	-0.05974600
C	-4.30907400	0.80483600	-0.20890400
C	-5.68222300	0.75160100	-0.40343300
H	-6.24993700	1.66922600	-0.52416500
C	-6.33580700	-0.49512900	-0.46094500
C	-5.59729000	-1.66952100	-0.33494700
C	-4.21419500	-1.61660200	-0.14374200
H	-7.40875400	-0.53022600	-0.61959700
H	-6.09150400	-2.63437900	-0.39326100
H	-3.63526600	-2.53024700	-0.06491700
H	-1.65798500	2.91051100	1.24791700
O	-1.41536400	-1.41243800	-0.09525400
C	-0.05302800	0.93944300	0.56202000
N	0.72729500	-0.13432800	0.24603600
B	0.04866000	-1.56550800	0.10015700
F	0.55673700	-2.19508900	-1.01799200
F	0.27823000	-2.29787100	1.25665600
C	4.06507600	1.37597400	-0.31835800
C	2.69567400	1.25719400	-0.19426200
C	2.09831000	0.00337600	0.11845600
C	2.94710200	-1.12569800	0.29745700
C	4.31622100	-1.00268000	0.17734400
C	4.87860300	0.24781400	-0.12736400
H	4.52023000	2.32308700	-0.57872800
H	2.07491000	2.12257500	-0.39477300
H	2.51737500	-2.07857600	0.57397600
H	4.96559800	-1.85524100	0.33005500
N	6.30751600	0.37568400	-0.25080200
O	6.78229000	1.50012800	-0.49570300
O	7.00682600	-0.64369700	-0.10816500
S	-3.46536700	2.38021800	-0.23279400
H	0.43974000	1.84759600	0.88839900
H	-2.79735300	1.77803900	2.01939800

Table S20. The T_1 state Cartesian coordinates of S-BF2 in CH_2Cl_2.

	X	Y	Z
C	-2.16288800	2.15453600	0.71758100
C	-1.40390800	0.95743200	0.28122000
C	-2.07438500	-0.25253500	-0.04458500
C	-3.49031000	-0.37783900	-0.10585100
C	-4.37775500	0.74126200	-0.05927500
C	-5.75738800	0.55321600	-0.13861600
H	-6.41219400	1.41687000	-0.08833300
C	-6.28903600	-0.71667400	-0.28223900
C	-5.43063700	-1.82773600	-0.33922800
C	-4.06825500	-1.66273200	-0.25132500
H	-7.36297800	-0.84992600	-0.34140700
H	-5.84474200	-2.82432600	-0.44655400
H	-3.40356200	-2.51632700	-0.29012100
H	-1.60058800	3.06961800	0.52663900
O	-1.35162400	-1.33510000	-0.31493200
C	-0.01615500	1.03100400	0.31127900
N	0.75078900	-0.06557000	0.11857500
B	0.03659600	-1.49527500	0.07454900
F	0.67000500	-2.27854500	-0.86997900
F	0.14589600	-2.04220100	1.34977500
C	4.11741400	1.32526000	-0.45525600
C	2.74379800	1.23443600	-0.39243300
C	2.12697200	0.04024000	0.03502400
C	2.93018900	-1.06442400	0.38867000
C	4.30318100	-0.96816800	0.33488600
C	4.88489700	0.22510900	-0.08466200
H	4.60450400	2.22766500	-0.79991700
H	2.14378200	2.07262600	-0.72347300
H	2.46382100	-1.97484400	0.73745300
H	4.93321200	-1.80008300	0.62007500
N	6.33735900	0.32396200	-0.14433400
O	6.82082000	1.38899100	-0.48256100
O	6.98626600	-0.66330700	0.14812500
S	-3.79262200	2.38768400	-0.02208900
H	0.46823300	1.97012000	0.54727200
H	-2.30877700	2.08101600	1.80500100

Table S21. The T_2 state Cartesian coordinates of S-BF2 in CH_2Cl_2.

	X	Y	Z
C	-2.21268800	2.00084500	1.09880900
C	-1.41005000	0.86526300	0.55338800
C	-2.07568700	-0.31794100	0.15704300
C	-3.49305600	-0.38549700	-0.04862600
C	-4.29982800	0.79427800	-0.16770600
C	-5.66482100	0.72636400	-0.49410900
H	-6.22846000	1.64764500	-0.59825400
C	-6.28328900	-0.50068900	-0.66531300
C	-5.50941400	-1.65880500	-0.55254700
C	-4.14232200	-1.60388000	-0.26491700
H	-7.33843100	-0.55689100	-0.89908500
H	-5.97007300	-2.62814100	-0.71027500
H	-3.55866300	-2.51528800	-0.22788100
H	-1.63584000	2.92412100	1.15699100
O	-1.38481000	-1.41505400	-0.09407800
C	-0.04125800	0.93284600	0.52518000
N	0.73152100	-0.14472500	0.23184300
B	0.05290400	-1.55294400	0.20535500
F	0.61059300	-2.33646700	-0.79501000
F	0.20690100	-2.16773300	1.45157500
C	4.03895500	1.35140700	-0.45328600
C	2.67199200	1.21746900	-0.32932800
C	2.10089400	-0.00203300	0.08531700
C	2.95538100	-1.09075100	0.35263900
C	4.32024900	-0.95943500	0.22393500
C	4.85898400	0.26271800	-0.17382400
H	4.48118100	2.28228800	-0.78355200
H	2.03688100	2.05359000	-0.59538600
H	2.53340800	-2.02820000	0.68754200
H	4.98194500	-1.78780900	0.44092400
N	6.29255600	0.40106300	-0.30517200
O	6.73892500	1.48931100	-0.63332200
O	6.98837500	-0.57647200	-0.08081500
S	-3.59532600	2.35606100	-0.02450000
H	0.45995600	1.85286600	0.80643600
H	-2.65138000	1.79178600	2.07776700

Table S22. The MECP1 Cartesian coordinates of S-BF2 in CH_2Cl_2.

	X	Y	Z
C	-2.24669573	2.01765582	0.95451439
C	-1.41525509	0.86858094	0.48641691
C	-2.07115543	-0.33842853	0.10826344
C	-3.49200317	-0.41569658	-0.08253972
C	-4.26067994	0.75409564	-0.29220365
C	-5.62719356	0.69000827	-0.49081480
H	-6.19382100	1.59966257	-0.65717072
C	-6.27349901	-0.55336027	-0.49473573
C	-5.53400515	-1.71458022	-0.31428942
C	-4.15848072	-1.65161380	-0.11878774
H	-7.34404056	-0.59991287	-0.65617230
H	-6.02573389	-2.68070000	-0.33495253
H	-3.57627232	-2.55796768	-0.00110994
H	-1.65785199	2.92704457	1.07174830
O	-1.37578454	-1.43547049	-0.05026092
C	-0.03927443	0.95473814	0.47566661
N	0.74423859	-0.12695876	0.21470400
B	0.07385094	-1.55323056	0.20621767
F	0.61045714	-2.31511736	-0.81459017
F	0.27002745	-2.15129828	1.44552592
C	4.05840670	1.37406589	-0.36705218
C	2.69362207	1.25114138	-0.25536645
C	2.10467147	0.00941713	0.08940513
C	2.95306326	-1.10467221	0.30654374
C	4.31706734	-0.97603736	0.19834037
C	4.86894152	0.26276669	-0.13374323
H	4.51338921	2.31434872	-0.64950614
H	2.07101901	2.10650559	-0.48533163
H	2.52431968	-2.05416914	0.59340325
H	4.97174509	-1.81815435	0.37999047
N	6.30098586	0.39698782	-0.24289310
O	6.75823210	1.50039389	-0.50964187
O	6.98830808	-0.59886980	-0.06324023
S	-3.42350031	2.30775250	-0.39325979
H	0.44471802	1.88127831	0.75901697
H	-2.79916897	1.81218797	1.87297707

Table S23. The MECP2 Cartesian coordinates of S-BF2 in CH_2Cl_2.

	X	Y	Z
C	-2.11785062	1.96207448	1.23271717
C	-1.44312852	0.78194837	0.64349560
C	-2.07977939	-0.31086976	0.10705042
C	-3.54301032	-0.37973014	-0.03406199
C	-4.37671365	0.73734925	0.21790656
C	-5.76064715	0.66466366	-0.04653009
H	-6.38583301	1.53103396	0.13492777
C	-6.30102998	-0.51009338	-0.51279931
C	-5.48619082	-1.62730413	-0.72225392
C	-4.11842377	-1.55410822	-0.49120898
H	-7.36433120	-0.57036086	-0.70891781
H	-5.91994095	-2.55416242	-1.07542999
H	-3.48021755	-2.40832466	-0.67353334
H	-1.61299972	2.89520465	0.96092184
O	-1.44777908	-1.32711143	-0.35170315
C	-0.01179015	0.78765521	0.61779365
N	0.68821505	-0.18853235	0.17888985
B	0.04413329	-1.52596405	-0.31858781
F	0.46173296	-1.79620526	-1.59296695
F	0.32798496	-2.52801278	0.57591588
C	4.04514279	0.88225275	-1.01389692
C	2.67735974	0.71844581	-0.93032584
C	2.12815197	-0.04522784	0.09887117
C	2.95095729	-0.67896891	1.02897864
C	4.32073620	-0.52052708	0.95088611
C	4.89317211	0.27252413	-0.06643154
H	4.49895141	1.47436973	-1.79595510
H	2.02569832	1.17881364	-1.66570623
H	2.51334037	-1.28921046	1.81115092
H	4.98662992	-0.99658428	1.65732479
N	6.26061055	0.45040973	-0.13576686
O	6.74837055	1.18031055	-1.08043828
O	7.01111518	-0.11731392	0.74551777
S	-3.83850580	2.23370848	0.84167667
H	0.52058792	1.67449299	0.95743137
H	-2.09589126	1.91790241	2.33383260

Table S24. The MECP3 Cartesian coordinates of S-BF2 in CH_2Cl_2.

	X	Y	Z
C	-2.17011441	2.11062199	0.89185446
C	-1.41190877	0.92131933	0.41761090
C	-2.07186087	-0.26481003	0.05799170
C	-3.50064804	-0.37638302	-0.07872019
C	-4.36904793	0.75707145	-0.06159460
C	-5.74933521	0.62003167	-0.25043701
H	-6.37564770	1.50536177	-0.23050717
C	-6.30801465	-0.63279170	-0.44942450
C	-5.47253753	-1.75106228	-0.46785197
C	-4.09707933	-1.62395062	-0.29097296
H	-7.37742452	-0.73711768	-0.58553951
H	-5.89602201	-2.73705073	-0.62502844
H	-3.45890829	-2.49759248	-0.32247114
H	-1.61181155	3.03521549	0.73903701
O	-1.37201844	-1.35666549	-0.20390174
C	-0.02873051	0.97976021	0.43353909
N	0.73825031	-0.11081663	0.20738403
B	0.03001036	-1.51705746	0.19782467
F	0.64059421	-2.34970484	-0.72468924
F	0.10021486	-2.05917811	1.48224507
C	4.07789404	1.30025533	-0.48264249
C	2.70796575	1.19680845	-0.38318816
C	2.10793629	0.00468524	0.07781213
C	2.93879328	-1.08521103	0.41963493
C	4.30721855	-0.97577235	0.33263842
C	4.87500916	0.21807368	-0.11432555
H	4.54552582	2.20052976	-0.85911706
H	2.09188550	2.02575983	-0.71041642
H	2.49271951	-1.99975991	0.78468771
H	4.95121160	-1.79803940	0.61638275
N	6.31047515	0.33469704	-0.20320093
O	6.77980716	1.38657437	-0.61408728
O	6.98948760	-0.62283353	0.13848164
S	-3.76776192	2.36879211	0.09541534
H	0.47060059	1.91077035	0.67952348
H	-2.39777602	2.03406092	1.96479838

Table S25. The MECP4 Cartesian coordinates of S-BF2 in CH_2Cl_2.

	X	Y	Z
C	-2.21860087	1.91017131	1.20496950
C	-1.40218946	0.81646123	0.57151835
C	-2.05983285	-0.37521747	0.19461221
C	-3.47860391	-0.43686910	-0.00586438
C	-4.24086464	0.76515104	-0.20553281
C	-5.60193138	0.78129610	-0.59784899
H	-6.07406062	1.73228771	-0.82431837
C	-6.31576656	-0.40971241	-0.67855000
C	-5.59235347	-1.57780750	-0.50785757
C	-4.19782816	-1.60462875	-0.19904988
H	-7.36111786	-0.42108293	-0.95117478
H	-6.08829013	-2.53160118	-0.65523152
H	-3.68822882	-2.55863439	-0.14943443
H	-1.67017903	2.84782785	1.30461283
O	-1.38300779	-1.47435301	-0.06543543
C	-0.05455806	0.87511131	0.50018623
N	0.72441489	-0.18940080	0.15646777
B	0.08757663	-1.57568544	0.20357493
F	0.60959268	-2.42449001	-0.77577214
F	0.23452945	-2.19061266	1.46536285
C	4.02879095	1.36301784	-0.50195379
C	2.65910313	1.19223309	-0.41025581
C	2.10340481	-0.01598060	0.03439951
C	2.98226004	-1.06207742	0.35459735
C	4.34782044	-0.90810407	0.23902626
C	4.87427761	0.30995153	-0.18207522
H	4.45079169	2.29967389	-0.84439054
H	2.01199068	2.00857133	-0.70982181
H	2.57804179	-2.00108718	0.70945268
H	5.02012854	-1.71739213	0.49344225
N	6.29857743	0.47583118	-0.29137516
O	6.73565864	1.56380559	-0.64264403
O	7.01929336	-0.47728356	-0.02829956
S	-3.53211626	2.27090901	0.01707725
H	0.46160848	1.79049899	0.78213977
H	-2.70212504	1.67038437	2.15320374

6.3 S-BF2*

Table S26. The S_0 state Cartesian coordinates of S-BF2* in CH_2Cl_2.

	X	Y	Z
C	-1.38478900	2.03147600	1.04796900
C	-0.54581400	0.89787400	0.55212400
C	-1.13613500	-0.29373500	0.15702600
C	-2.58033200	-0.43607200	0.00678800
C	-3.42131800	0.68952000	-0.11696700
C	-4.77794300	0.50459700	-0.38100700
H	-5.42408600	1.36992000	-0.48145600
C	-5.30349400	-0.77360600	-0.49647000
C	-4.48466400	-1.89133500	-0.34750300
C	-3.13452400	-1.71826900	-0.10527100
H	-6.36346700	-0.89719600	-0.69109800
H	-4.90046300	-2.88933400	-0.42381600
H	-2.47693600	-2.57311600	-0.00165500
H	-0.81689700	2.96294100	1.06841300
O	-0.42409300	-1.33110900	-0.17299000
C	0.84426900	1.00569700	0.45912900
N	1.63125400	-0.00722600	0.16319500
B	1.03269900	-1.44714200	0.07644300
F	1.59772800	-2.14142400	-0.97227800
F	1.23071200	-2.10762300	1.28571500
C	4.84005000	1.51440600	-0.91499900
C	3.47587100	1.31137200	-0.74853000
C	3.02211800	0.20371100	-0.03407800
C	3.93403400	-0.71331800	0.48841000
C	5.29389200	-0.50553200	0.30847800
H	5.18679600	2.37609400	-1.47503800
H	2.76429400	1.99551000	-1.19862300
H	3.57037900	-1.56811600	1.04564600
H	6.00003000	-1.21768900	0.72160500
S	-2.80723100	2.33929300	-0.03842000
H	1.31484200	1.97044000	0.63561000
H	-1.76563100	1.84132200	2.05521300
C	5.75282400	0.60855400	-0.38781500
H	6.81708600	0.76508100	-0.52537800

Table S27. The S_1 state Cartesian coordinates of S-BF2* in CH_2Cl_2.

	X	Y	Z
C	-1.30263600	2.18283600	0.64848600
C	-0.52076800	0.98602200	0.25802500
C	-1.14005700	-0.25471200	-0.00771700
C	-2.55292000	-0.44031800	-0.08946900
C	-3.49062000	0.64218400	-0.03963200
C	-4.86562000	0.41390900	-0.13584100
H	-5.54668100	1.25712800	-0.08403600
C	-5.35366100	-0.87221600	-0.29517100
C	-4.45119000	-1.94263200	-0.35731600
C	-3.08787200	-1.73309600	-0.25613800
H	-6.42084000	-1.04422600	-0.36700400
H	-4.82492000	-2.95293300	-0.48481600
H	-2.39769900	-2.56537700	-0.30475500
H	-0.80869100	3.10724900	0.33997700
O	-0.38879700	-1.32615300	-0.22480600
C	0.86002300	1.09454900	0.25866800
N	1.66899700	0.02880200	0.08633700
B	1.02996800	-1.40122000	0.13810300
F	1.65400700	-2.23763000	-0.77649400
F	1.16076700	-1.91049600	1.43586100
C	4.95462000	1.55072500	-0.68540200
C	3.58236200	1.39084500	-0.58063700
C	3.04231300	0.20394900	-0.05328300
C	3.92225200	-0.81932400	0.34574400
C	5.28993100	-0.64540400	0.23231600
H	5.35142200	2.46902500	-1.10560500
H	2.92473600	2.16998500	-0.94917200
H	3.51836300	-1.72926500	0.76940000
H	5.95309500	-1.44092000	0.55573500
S	-2.99023500	2.29670200	0.03731200
H	1.32151300	2.05894300	0.44326000
H	-1.41022900	2.22932600	1.74464700
C	5.81972700	0.53834000	-0.27918000
H	6.89299100	0.66673600	-0.36607000

Table S28. The S_2 state Cartesian coordinates of S-BF2* in CH_2Cl_2.

	X	Y	Z
C	-1.39148600	2.01921000	1.00945600
C	-0.53729600	0.90832700	0.47778900
C	-1.15826500	-0.29302600	0.06834200
C	-2.58866600	-0.44007000	-0.03472400
C	-3.43661000	0.69012000	-0.13814000
C	-4.81163900	0.57638400	-0.25383000
H	-5.42220100	1.46815500	-0.34675500
C	-5.40423500	-0.69388100	-0.26932500
C	-4.59138800	-1.81584900	-0.20526500
C	-3.20236300	-1.69574000	-0.10252300
H	-6.47921200	-0.78820800	-0.36148800
H	-5.03175400	-2.80602300	-0.25120800
H	-2.57564600	-2.57966100	-0.09121300
H	-0.84136700	2.95667900	1.08944800
O	-0.42930700	-1.36095900	-0.18971600
C	0.83346100	1.03536100	0.44526400
N	1.64119300	-0.00645000	0.11343400
B	1.02750900	-1.43458500	0.05392200
F	1.58624900	-2.15723800	-0.99278300
F	1.24152500	-2.09798600	1.26942300
C	4.89471800	1.62221700	-0.57101300
C	3.52727100	1.43607200	-0.44559000
C	3.01122700	0.19627000	-0.02804400
C	3.91216700	-0.84943700	0.24572100
C	5.27471300	-0.65121700	0.10783300
H	5.27134900	2.58390800	-0.90385000
H	2.85144400	2.24168300	-0.70966400
H	3.52851800	-1.80156400	0.58815500
H	5.95347500	-1.46830400	0.32932800
S	-2.67770500	2.27125400	-0.23482300
H	1.30222000	1.96273700	0.75541100
H	-1.87584900	1.79108500	1.96139100
C	5.78028900	0.58356500	-0.29587400
H	6.84951000	0.73240000	-0.39852200

Table S29. The T_1 state Cartesian coordinates of S-BF2* in CH_2Cl_2.

	X	Y	Z
C	-1.35410800	2.11121400	0.79594700
C	-0.51857100	0.96959700	0.32950800
C	-1.15539400	-0.26861200	0.00543900
C	-2.57200600	-0.43671400	-0.05528300
C	-3.47139600	0.66380400	-0.09532500
C	-4.84224300	0.45167700	-0.16888300
H	-5.51042700	1.30655800	-0.18569000
C	-5.35794600	-0.83872300	-0.23110800
C	-4.48829100	-1.93241000	-0.20601600
C	-3.12482600	-1.73643700	-0.11778700
H	-6.42992700	-0.98879100	-0.29222500
H	-4.88392800	-2.94149100	-0.24982900
H	-2.44598800	-2.58011200	-0.09568000
H	-0.80944300	3.05382500	0.73450300
O	-0.39745500	-1.33786600	-0.24869800
C	0.84745000	1.11192400	0.32249200
N	1.67108200	0.03702700	0.08622800
B	1.00465800	-1.42953200	0.08313300
F	1.63371700	-2.19337300	-0.88261500
F	1.20490700	-1.94761000	1.35860800
C	4.94571800	1.60692000	-0.50267200
C	3.58004000	1.46376000	-0.38813000
C	3.02319400	0.20726200	-0.03809200
C	3.89347500	-0.88829600	0.19426300
C	5.25708100	-0.71761500	0.09289500
H	5.35988200	2.56471000	-0.79582600
H	2.93373800	2.29969300	-0.62280700
H	3.47974200	-1.84180100	0.48860000
H	5.91499000	-1.55554200	0.29228100
S	-2.87295300	2.32163500	-0.17201300
H	1.29955800	2.05825400	0.58485000
H	-1.63665600	1.94525700	1.84174900
C	5.79430800	0.52380700	-0.25713000
H	6.86795900	0.64658200	-0.34203400

Table S30. The T_2 state Cartesian coordinates of S-BF2* in CH_2Cl_2.

	X	Y	Z
C	-1.36075800	2.02822000	1.02823000
C	-0.52617600	0.91322000	0.49416900
C	-1.14462200	-0.30895800	0.14342100
C	-2.56087200	-0.45337500	-0.00893300
C	-3.44563800	0.68232300	-0.10184000
C	-4.82035000	0.52903800	-0.35686300
H	-5.44019500	1.41596400	-0.43939600
C	-5.37361300	-0.73003900	-0.48760900
C	-4.52183400	-1.84364700	-0.40955300
C	-3.15140200	-1.70854200	-0.19016800
H	-6.43392300	-0.85265500	-0.66549800
H	-4.93222800	-2.83939000	-0.54071300
H	-2.51545600	-2.58502100	-0.17553700
H	-0.82887400	2.98012300	1.02618100
O	-0.40774300	-1.37766100	-0.11040300
C	0.83960700	1.03731000	0.41826700
N	1.64749100	-0.00485100	0.12950900
B	1.04483200	-1.43681800	0.15315600
F	1.61750300	-2.22333500	-0.83839900
F	1.25231900	-2.02131200	1.41136900
C	4.85926000	1.58871300	-0.81574200
C	3.49594500	1.38737800	-0.64792700
C	3.02020900	0.20672600	-0.06291100
C	3.94220200	-0.77325400	0.32959600
C	5.30033200	-0.56620900	0.14585000
H	5.20801400	2.50758100	-1.27581800
H	2.79468500	2.13524400	-1.00223700
H	3.58184000	-1.68098400	0.79688900
H	6.00036700	-1.33363200	0.45993500
S	-2.81835600	2.27907400	-0.03612900
H	1.31037800	1.99049300	0.64057400
H	-1.74924700	1.84144800	2.03297200
C	5.77106300	0.61524300	-0.42205100
H	6.83516700	0.77192300	-0.56075700

6.4 S-BF2_C

Table S31. The S_0 state Cartesian coordinates of S-BF2_C in CH_2Cl_2.

	X	Y	Z
C	-3.23288800	1.93435300	1.23734000
C	-2.39612300	0.84993800	0.63835200
C	-2.99030400	-0.28890900	0.11579000
C	-4.43315900	-0.40179800	-0.06771600
C	-5.26383300	0.73803900	-0.08524200
C	-6.61822500	0.59354100	-0.38349700
H	-7.25644500	1.47029600	-0.40164500
C	-7.15197600	-0.66110800	-0.63744700
C	-6.34367500	-1.79544200	-0.59492300
C	-4.99564300	-1.66046300	-0.31881400
H	-8.21018100	-0.75440000	-0.85698100
H	-6.76591300	-2.77645500	-0.77954100
H	-4.34597700	-2.52724900	-0.29572700
H	-2.65749100	2.85302300	1.36223000
O	-2.28123700	-1.29418500	-0.30879900
C	-1.00356800	0.95374000	0.57707800
N	-0.21995200	-0.03140800	0.19133100
B	-0.83152800	-1.44962800	-0.04535800
F	-0.25313700	-2.04731400	-1.14508700
F	-0.66219500	-2.22534500	1.09889700
C	3.01131100	1.58399900	-0.68087200
C	1.64774100	1.36174100	-0.54539600
C	1.17491900	0.18153900	0.03516200
C	2.06441400	-0.80757100	0.46403800
C	3.42029600	-0.58601500	0.31919700
C	3.90160700	0.60751900	-0.24592600
H	3.36405800	2.50244800	-1.13816500
H	0.94221300	2.09481800	-0.92149600
H	1.68178200	-1.71500600	0.91649600
S	-4.63768100	2.36544400	0.17009500
H	-0.52863900	1.89125200	0.85737600
H	-3.62933900	1.64462300	2.21442600
C	5.36227100	0.55656000	-0.26474300
C	5.77052700	-0.66651200	0.29297600
C	4.57034000	-1.47667200	0.70185100

H	4.57271600	-1.70171500	1.77452400
H	4.52810800	-2.43931600	0.17952900
C	6.30240400	1.47752600	-0.71990500
C	7.65221100	1.16295800	-0.61120800
C	8.05752100	-0.05127000	-0.05700100
C	7.11798900	-0.97394800	0.39873700
H	5.99327900	2.42361000	-1.15274200
H	8.39878800	1.86837200	-0.96096300
H	9.11563800	-0.27912200	0.01927500
H	7.44054100	-1.91733900	0.82847900

Table S32. The S_1 state Cartesian coordinates of S-BF2_C in CH_2Cl_2.

	X	Y	Z
C	-3.24149800	2.07207700	0.97529600
C	-2.38704600	0.95635900	0.48350800
C	-2.99681600	-0.27535700	0.09731000
C	-4.39661700	-0.42190600	-0.10556300
C	-5.29086000	0.69139800	-0.16351800
C	-6.64492100	0.48922900	-0.41703400
H	-7.30921500	1.34687700	-0.44869300
C	-7.14688500	-0.78728300	-0.62659700
C	-6.28499200	-1.89083700	-0.57415800
C	-4.94408100	-1.71295600	-0.32061300
H	-8.20474700	-0.92513900	-0.82041600
H	-6.67541600	-2.89071200	-0.73058500
H	-4.27196500	-2.56108800	-0.28038400
H	-2.69900900	3.01837100	0.98115000
O	-2.24343900	-1.33993900	-0.10092300
C	-1.02738100	1.07890500	0.50922500
N	-0.18636000	0.02371000	0.23925000
B	-0.80673800	-1.41443400	0.19238900
F	-0.21614100	-2.15120000	-0.82773000
F	-0.61232800	-2.04390200	1.42432900
C	3.03834700	1.72303300	-0.33075600
C	1.68653300	1.51710400	-0.20408400
C	1.15804000	0.22843700	0.10674100
C	2.05908600	-0.85903600	0.28162500
C	3.40339000	-0.64188200	0.15646100
C	3.91861900	0.64519900	-0.14747000
H	3.41197600	2.70893600	-0.58546200

H	1.00914700	2.33980700	-0.39422600
H	1.67045300	-1.83442300	0.53998300
S	-4.72740700	2.34465700	-0.03546400
H	-0.57762900	2.01271800	0.82559400
H	-3.59080200	1.87026600	1.99430300
C	5.35250700	0.57100600	-0.20980900
C	5.74696000	-0.76110600	0.05979000
C	4.53780500	-1.61670100	0.31327700
H	4.55541700	-2.06378300	1.31333800
H	4.46446300	-2.44405200	-0.40106600
C	6.30974300	1.55862500	-0.47880500
C	7.64677300	1.20141600	-0.47554400
C	8.03325300	-0.11741600	-0.20838800
C	7.08561600	-1.10532800	0.06057400
H	6.01156700	2.58059900	-0.68660000
H	8.40587700	1.94796200	-0.68189200
H	9.08734200	-0.37364100	-0.21100400
H	7.39954000	-2.12347800	0.26586600

Table S33. The S_2 state Cartesian coordinates of S-BF2_C in CH_2Cl_2.

	X	Y	Z
C	-3.22322900	2.03721900	1.03268600
C	-2.38514800	0.92551900	0.48289500
C	-2.99111800	-0.24717900	0.02081500
C	-4.42378800	-0.41404500	-0.09749000
C	-5.31646300	0.69022600	-0.08630700
C	-6.69048600	0.53237500	-0.24255600
H	-7.33183300	1.40738600	-0.24164300
C	-7.23529600	-0.74397200	-0.40660800
C	-6.37803100	-1.83390600	-0.43775200
C	-4.99279200	-1.67154300	-0.29481300
H	-8.30431000	-0.86879200	-0.52537100
H	-6.77616200	-2.83155700	-0.58854800
H	-4.33535100	-2.53037800	-0.35431600
H	-2.68643100	2.98652300	1.04058700
O	-2.25504900	-1.29939000	-0.31471000
C	-1.01102200	1.04022800	0.50001200
N	-0.19877100	0.01195600	0.13945600
B	-0.82022400	-1.40403200	-0.00467900
F	-0.21214400	-2.09784500	-1.04731500

F	-0.66197500	-2.11976300	1.19532300
C	3.05567800	1.67812200	-0.47794600
C	1.69256700	1.46405900	-0.37871600
C	1.17616100	0.21731300	0.03092600
C	2.06512700	-0.83187100	0.33134100
C	3.42094800	-0.61836400	0.21590400
C	3.93300300	0.63430500	-0.18082900
H	3.42684600	2.64547300	-0.80097400
H	1.00699500	2.25807200	-0.65256100
H	1.67031700	-1.78349900	0.66469900
S	-4.68135800	2.31094600	0.00162400
H	-0.54449300	1.95389000	0.85303700
H	-3.59401300	1.83579400	2.04379100
C	5.38315800	0.56152100	-0.19747100
C	5.77046600	-0.73707900	0.19263000
C	4.55343300	-1.57396800	0.47914700
H	4.54497000	-1.94530000	1.51019500
H	4.50063300	-2.45409100	-0.17179000
C	6.34555600	1.52131400	-0.51774800
C	7.68684100	1.17147200	-0.44470600
C	8.06836000	-0.11524500	-0.05729400
C	7.11003300	-1.07678700	0.26415900
H	6.05457700	2.52247100	-0.81881300
H	8.44868000	1.90380600	-0.69055100
H	9.12207100	-0.36871800	-0.00654200
H	7.41547100	-2.07444800	0.56395000

Table S34. The T_1 state Cartesian coordinates of S-BF2_C in CH_2Cl_2.

	X	Y	Z
C	-3.25077300	2.09067300	0.88683400
C	-2.38384900	0.97452700	0.40232200
C	-2.99618200	-0.24941900	0.02365700
C	-4.41506400	-0.42827900	-0.08909100
C	-5.31296300	0.66778300	-0.14846800
C	-6.67892800	0.45429100	-0.28334600
H	-7.34836000	1.30783000	-0.31645000
C	-7.18810000	-0.83688000	-0.38426600
C	-6.31847100	-1.92643800	-0.33621800
C	-4.95817900	-1.72582200	-0.18944300
H	-8.25625400	-0.98832600	-0.49310500

H	-6.70757000	-2.93646900	-0.40937000
H	-4.27742200	-2.56769200	-0.15254600
H	-2.71200300	3.03883000	0.88996900
O	-2.22620200	-1.30869400	-0.24782400
C	-1.02425200	1.13197500	0.42847300
N	-0.16980300	0.06993800	0.16090500
B	-0.82888900	-1.39782500	0.09776000
F	-0.18819600	-2.12951700	-0.89104400
F	-0.63524500	-1.96860100	1.35358100
C	3.07145900	1.74801100	-0.25421300
C	1.71862300	1.56038000	-0.15735300
C	1.16316500	0.25616600	0.06164900
C	2.04993600	-0.86089400	0.18804700
C	3.39593200	-0.65420300	0.10972900
C	3.93102800	0.64379700	-0.11213400
H	3.46690800	2.73846100	-0.44929100
H	1.05833700	2.40359300	-0.30799700
H	1.64365700	-1.84346200	0.37900500
S	-4.70968900	2.32949100	-0.16500700
H	-0.58952100	2.06591600	0.75402300
H	-3.59297700	1.88399500	1.90601900
C	5.36834200	0.55428900	-0.14927700
C	5.73925300	-0.79200400	0.05403000
C	4.51737400	-1.64794900	0.23681600
H	4.50885500	-2.14673200	1.21206900
H	4.45015000	-2.43507600	-0.52195200
C	6.33762600	1.54427900	-0.34376300
C	7.67168300	1.17261800	-0.33326400
C	8.03761500	-0.16253700	-0.13195100
C	7.07568400	-1.15213500	0.06250200
H	6.05410200	2.57941000	-0.50072900
H	8.44213100	1.92099400	-0.48252500
H	9.08874300	-0.43064900	-0.12748200
H	7.37414800	-2.18367000	0.21745600

Table S35. The T_2 state Cartesian coordinates of S-BF2_C in CH_2Cl_2.

	X	Y	Z
C	-3.21663900	2.00644000	1.10165100
C	-2.38538700	0.90779000	0.52769200
C	-2.99457700	-0.27989500	0.08291100

C	-4.40963100	-0.42138400	-0.09302600
C	-5.31187000	0.70670800	-0.08249500
C	-6.68605300	0.55124100	-0.34469300
H	-7.32247200	1.43020500	-0.33917500
C	-7.21619800	-0.69746200	-0.59240900
C	-6.34563600	-1.80312400	-0.61411900
C	-4.98052300	-1.66577200	-0.38142300
H	-8.27509400	-0.82232100	-0.77787700
H	-6.74185100	-2.78967600	-0.83049200
H	-4.33034200	-2.53007900	-0.43507000
H	-2.68459300	2.95860100	1.11465000
O	-2.25383700	-1.32780100	-0.24389200
C	-1.01402000	1.02373400	0.50667100
N	-0.20045700	-0.00177200	0.17339400
B	-0.81682200	-1.42571200	0.06762300
F	-0.21270800	-2.14318700	-0.95796700
F	-0.65696500	-2.10412600	1.28598900
C	3.03926300	1.65540300	-0.55039200
C	1.67702300	1.43694500	-0.43055800
C	1.17499600	0.20772800	0.03796200
C	2.07202800	-0.82299200	0.37177200
C	3.42628300	-0.60469400	0.23850900
C	3.92577400	0.63253300	-0.21475500
H	3.40023500	2.60936100	-0.92138200
H	0.98461900	2.21317700	-0.73688900
H	1.68676400	-1.76287900	0.74757800
S	-4.71657100	2.30496500	0.11590900
H	-0.54682400	1.95685500	0.80691700
H	-3.56973900	1.79284400	2.11537500
C	5.37959300	0.56745000	-0.24128800
C	5.77745800	-0.70899400	0.20045600
C	4.56783500	-1.54034800	0.53399400
H	4.56986600	-1.86555400	1.58065100
H	4.51536300	-2.44909800	-0.07653100
C	6.33296800	1.51644200	-0.61072300
C	7.67848400	1.17771900	-0.53435500
C	8.07135000	-0.08755300	-0.09557800
C	7.12032700	-1.03852300	0.27484400
H	6.03429500	2.50226600	-0.95275400
H	8.43294100	1.90402100	-0.81857200
H	9.12685900	-0.33327200	-0.04276300

H	7.43309900	-2.02106600	0.61495700

6.5 S-BF2_N

Table S36. The S_0 state Cartesian coordinates of S-BF2_N in CH_2Cl_2.

	X	Y	Z
C	-3.22585500	1.94158900	1.21785800
C	-2.38650800	0.85699500	0.62260600
C	-2.97636900	-0.29108000	0.11702900
C	-4.42022400	-0.41758200	-0.05068900
C	-5.26011000	0.71535100	-0.07182100
C	-6.61630500	0.55709400	-0.35446600
H	-7.26150100	1.42867700	-0.37549300
C	-7.14268900	-0.70437000	-0.58948000
C	-6.32492600	-1.83171600	-0.54321500
C	-4.97516800	-1.68315700	-0.28257800
H	-8.20229200	-0.80831900	-0.79714200
H	-6.74113600	-2.81796100	-0.71307000
H	-4.31839700	-2.54452100	-0.25719800
H	-2.65638200	2.86594400	1.32689600
O	-2.26363000	-1.29496100	-0.30509700
C	-0.99466200	0.96920700	0.55158100
N	-0.20682900	-0.01263400	0.16762500
B	-0.80857600	-1.43528300	-0.06434900
F	-0.24205300	-2.02596600	-1.17428100
F	-0.61382700	-2.21347600	1.07516400
C	2.99992100	1.64007300	-0.72525100
C	1.64401900	1.39942600	-0.58331500
C	1.18772900	0.21045200	0.01062700
C	2.07303600	-0.76768700	0.44952000
C	3.43015800	-0.51712500	0.29064400
C	3.91345300	0.68328000	-0.28567200
H	3.34034900	2.55844900	-1.19167500
H	0.92514900	2.11559100	-0.96516200
H	1.70556500	-1.67475800	0.91296100
S	-4.64391600	2.35023400	0.15951500
H	-0.52396000	1.91140700	0.82295700
H	-3.61068000	1.65994600	2.20196700
C	5.35269700	0.59291200	-0.27850400
C	5.67298500	-0.65606600	0.30457900

C	6.38043100	1.43738800	-0.70248400
C	7.69466800	1.02823900	-0.54072600
C	7.99290900	-0.21352100	0.03847300
C	6.99226600	-1.07193900	0.46854200
H	6.15369200	2.39880200	-1.15190900
H	8.50410000	1.67308700	-0.86515200
H	9.02997100	-0.51116600	0.15327300
H	7.22826600	-2.03173100	0.91519300
N	4.50300400	-1.30045400	0.63864500
H	4.44278400	-2.21106800	1.06246800

Table S37. The S_1 state Cartesian coordinates of S-BF2_N in CH_2Cl_2.

	X	Y	Z
C	-3.24806900	2.07002900	0.96585500
C	-2.38411300	0.96169400	0.47415400
C	-2.98311100	-0.27871600	0.09735000
C	-4.38208200	-0.44160500	-0.09503100
C	-5.28864900	0.66191700	-0.15360800
C	-6.64233200	0.44387200	-0.39434800
H	-7.31578500	1.29437600	-0.42618300
C	-7.13293300	-0.83927600	-0.59174400
C	-6.25892600	-1.93328100	-0.53956200
C	-4.91803400	-1.73987200	-0.29775400
H	-8.19084800	-0.98936200	-0.77597300
H	-6.63997900	-2.93819300	-0.68682100
H	-4.23685500	-2.58075500	-0.25753700
H	-2.71561000	3.02206200	0.96575500
O	-2.21922300	-1.33705600	-0.09760600
C	-1.02582400	1.09804100	0.49275700
N	-0.17557300	0.05158400	0.22095900
B	-0.77888700	-1.39227200	0.17751400
F	-0.18890500	-2.12034600	-0.85007800
F	-0.56145200	-2.02464300	1.40558000
C	3.02497700	1.79032200	-0.36871600
C	1.67924700	1.56541600	-0.23906800
C	1.16847500	0.27010500	0.08756900
C	2.06923400	-0.80981100	0.27743100
C	3.41273600	-0.56079800	0.14136500
C	3.92974200	0.73039400	-0.17407200
H	3.38572800	2.77742300	-0.63634700

H	0.98825000	2.37301500	-0.44173900
H	1.69649800	-1.78666200	0.55086200
S	-4.74008600	2.32200100	-0.04257600
H	-0.58361200	2.03751900	0.80273800
H	-3.59246200	1.86893200	1.98657600
C	5.34345700	0.61255400	-0.21838700
C	5.64708900	-0.75259200	0.07480600
C	6.39017400	1.51429900	-0.47419200
C	7.68757500	1.05057100	-0.43708600
C	7.96527500	-0.30182100	-0.14758700
C	6.95885900	-1.21738600	0.11082900
H	6.17734800	2.55409300	-0.69704600
H	8.51072800	1.72846300	-0.63225500
H	8.99767400	-0.63400500	-0.12671900
H	7.18302200	-2.25453400	0.33209300
N	4.47588200	-1.42591300	0.28827300
H	4.39993500	-2.40570900	0.50720400

Table S38. The S_2 state Cartesian coordinates of S-BF2_N in CH_2Cl_2.

	X	Y	Z
C	-3.28187300	2.03323700	1.02861000
C	-2.40694200	0.94965600	0.48115300
C	-2.98930000	-0.25464600	0.04866500
C	-4.40490800	-0.44204200	-0.09430500
C	-5.31611000	0.64718600	-0.11149500
C	-6.67601600	0.42718600	-0.28968500
H	-7.35263400	1.27611100	-0.29058300
C	-7.17289600	-0.86032100	-0.47299900
C	-6.29198600	-1.94182600	-0.46547400
C	-4.93746100	-1.73640600	-0.27879100
H	-8.23701000	-1.01431200	-0.61358100
H	-6.66808000	-2.95047600	-0.60278200
H	-4.24893500	-2.57285700	-0.27417800
H	-2.75052800	2.98569900	1.07053700
O	-2.21543800	-1.29764100	-0.26938500
C	-1.04216600	1.09323900	0.49583200
N	-0.20068000	0.06302000	0.16283100
B	-0.79464100	-1.36121900	0.04706700
F	-0.14849200	-2.06566700	-0.97713600
F	-0.60014400	-2.05996500	1.26072800

C	3.05832600	1.78095400	-0.47322800
C	1.68081500	1.52660200	-0.35497900
C	1.16297500	0.28081900	0.05607600
C	2.06061900	-0.75184600	0.34362700
C	3.42277900	-0.48964800	0.18862000
C	3.94924200	0.76911100	-0.20620000
H	3.39210300	2.76155100	-0.79217900
H	0.99003400	2.31845300	-0.61767500
H	1.70366800	-1.71503200	0.68288100
S	-4.73649700	2.31556300	-0.02543200
H	-0.59589000	2.01940300	0.84261500
H	-3.63962300	1.79472200	2.03482800
C	5.39867200	0.62451800	-0.22575600
C	5.66678800	-0.71064600	0.16070100
C	6.45562100	1.45817100	-0.51874400
C	7.76343600	0.94530500	-0.42340400
C	8.00689700	-0.37031100	-0.04395100
C	6.95242800	-1.23003400	0.25778600
H	6.29511300	2.48722900	-0.81814400
H	8.59965200	1.59518300	-0.65343800
H	9.02547500	-0.73315400	0.01792300
H	7.12468200	-2.25760300	0.55433200
N	4.46567300	-1.33639500	0.39906000
H	4.37176700	-2.30193200	0.68820300

Table S39. The T_1 state Cartesian coordinates of S-BF2_N in CH_2Cl_2.

	X	Y	Z
C	-3.25242000	2.07529500	0.90691000
C	-2.37714100	0.96999200	0.41483200
C	-2.98242700	-0.25901300	0.02771900
C	-4.39768300	-0.44403100	-0.09383500
C	-5.30292600	0.64766100	-0.14640700
C	-6.66694600	0.42747700	-0.28744200
H	-7.34104400	1.27759500	-0.31430700
C	-7.16877700	-0.86552700	-0.40337400
C	-6.29239600	-1.95052900	-0.36393200
C	-4.93413400	-1.74414300	-0.21001200
H	-8.23562700	-1.02198100	-0.51743500
H	-6.67539000	-2.96196700	-0.44936700
H	-4.24886400	-2.58256300	-0.17947900

H	-2.72024900	3.02702800	0.92138600
O	-2.20357200	-1.31418200	-0.23751400
C	-1.01993400	1.13606600	0.44197500
N	-0.16015700	0.08207000	0.16275200
B	-0.80612400	-1.38999800	0.11089600
F	-0.15788600	-2.12668900	-0.86940500
F	-0.60942900	-1.94935200	1.37214300
C	3.05002500	1.80759200	-0.30278200
C	1.70378000	1.59692400	-0.19629400
C	1.17367300	0.28585800	0.05219900
C	2.06126900	-0.81894200	0.19704900
C	3.40970200	-0.57572400	0.10395900
C	3.93841100	0.72571300	-0.14157300
H	3.42827900	2.79950400	-0.52232800
H	1.02484500	2.42108800	-0.36614700
H	1.67462500	-1.80455200	0.40937300
S	-4.71023600	2.31314600	-0.14721600
H	-0.58857800	2.06946500	0.77411400
H	-3.59675800	1.85548900	1.92267300
C	5.35525300	0.59863500	-0.16890000
C	5.64269700	-0.77500600	0.06402000
C	6.40756700	1.50571200	-0.36584300
C	7.70319000	1.03378700	-0.32966200
C	7.96717900	-0.33011500	-0.09943300
C	6.95157000	-1.25014500	0.09942200
H	6.20320100	2.55590400	-0.54364400
H	8.53229100	1.71575800	-0.47984500
H	8.99690800	-0.67049100	-0.07714000
H	7.16632700	-2.29779800	0.27546600
N	4.46226500	-1.45206000	0.22754300
H	4.37688300	-2.44030400	0.39948100

Table S40. The T_2 state Cartesian coordinates of S-BF2_N in CH_2Cl_2.

	X	Y	Z
C	-3.21456400	1.99870400	1.10502700
C	-2.37752500	0.90663000	0.52732300
C	-2.98241600	-0.28771400	0.09037000
C	-4.39702700	-0.43733500	-0.07879200
C	-5.30459400	0.68725800	-0.07191000
C	-6.67907500	0.52519900	-0.32830400

H	-7.31849100	1.40202800	-0.32679300
C	-7.20582800	-0.72714900	-0.56565800
C	-6.33027900	-1.82893900	-0.58562700
C	-4.96431100	-1.68509500	-0.35983200
H	-8.26491900	-0.85727800	-0.74607500
H	-6.72299400	-2.81819200	-0.79611100
H	-4.31100500	-2.54703500	-0.41386800
H	-2.68829500	2.95395000	1.12134900
O	-2.23692300	-1.33307700	-0.23315000
C	-1.00807000	1.03238900	0.49522500
N	-0.18989200	0.01352700	0.15384900
B	-0.79285000	-1.41484500	0.05144200
F	-0.19989300	-2.12294300	-0.98742900
F	-0.60481100	-2.09927800	1.26374000
C	3.02272500	1.71098100	-0.59646400
C	1.66843400	1.47079600	-0.46942600
C	1.18648300	0.23494800	0.02018800
C	2.08177200	-0.78072900	0.37105800
C	3.43725700	-0.53032700	0.22130900
C	3.93463300	0.71063000	-0.24907000
H	3.36868600	2.66404600	-0.98318200
H	0.96020000	2.22697800	-0.78788400
H	1.71451000	-1.71949600	0.76504000
S	-4.71238100	2.28800000	0.11175400
H	-0.54479900	1.96844800	0.79218100
H	-3.57056100	1.78016600	2.11646800
C	5.36926800	0.60694800	-0.25632500
C	5.68081800	-0.69398100	0.21463500
C	6.40598700	1.47505600	-0.60899100
C	7.71654200	1.04044800	-0.48967900
C	8.00481900	-0.25027100	-0.02250100
C	6.99591200	-1.13376700	0.33555100
H	6.18768900	2.47453400	-0.97124500
H	8.53132000	1.70340500	-0.75999100
H	9.03922300	-0.56693200	0.06129300
H	7.22435700	-2.13106300	0.69574500
N	4.50586000	-1.35273900	0.49477900
H	4.43761300	-2.29630300	0.83716400

6.6 S-BF2_O

Table S41. The S_0 state Cartesian coordinates of S-BF2_O in CH_2Cl_2.

	X	Y	Z
C	-3.23017400	1.94055500	1.21580000
C	-2.38137700	0.86173000	0.62388700
C	-2.96282900	-0.29494000	0.12385800
C	-4.40506400	-0.43206600	-0.04466000
C	-5.25268000	0.69518500	-0.07245200
C	-6.60690100	0.52612300	-0.35853400
H	-7.25822000	1.39297700	-0.38496700
C	-7.12359400	-0.73994100	-0.58955500
C	-6.29829000	-1.86164100	-0.53623900
C	-4.95050200	-1.70271200	-0.27285000
H	-8.18188000	-0.85216000	-0.79959700
H	-6.70739800	-2.85137100	-0.70284200
H	-4.28788700	-2.55939000	-0.24206800
H	-2.66846700	2.86962500	1.32449100
O	-2.24270400	-1.29516500	-0.29148800
C	-0.99259100	0.98629000	0.54820900
N	-0.19706200	0.00813700	0.16596900
B	-0.78578700	-1.42265400	-0.05304400
F	-0.21507000	-2.01337500	-1.16017400
F	-0.58058400	-2.18899000	1.09094800
C	2.99314100	1.68104600	-0.75805500
C	1.63981600	1.42611500	-0.60374600
C	1.19244900	0.24106500	0.00028200
C	2.09015800	-0.73020400	0.44146000
C	3.42971900	-0.45292600	0.26453000
C	3.90992200	0.73012800	-0.31575200
H	3.32343800	2.59841500	-1.23204000
H	0.91354200	2.13533600	-0.98450100
H	1.74840000	-1.64217900	0.91311600
S	-4.64989700	2.33528600	0.15448300
H	-0.52943600	1.93363000	0.81462100
H	-3.61436200	1.65676600	2.19947900
C	5.34922600	0.60842600	-0.29562300
C	5.60469500	-0.63414600	0.29996100
C	6.42178100	1.40017200	-0.70601800
C	7.70908100	0.92186800	-0.50674200
C	7.93341000	-0.32397700	0.09113300
C	6.87913200	-1.12885500	0.50760400

H	6.25460500	2.36614900	-1.16991900
H	8.55758600	1.52086800	-0.81857600
H	8.95108400	-0.67106000	0.23316500
H	7.03996500	-2.09494800	0.97080800
O	4.44714000	-1.27901000	0.64081300

Table S42. The S_1 state Cartesian coordinates of S-BF2_O in CH_2Cl_2.

	X	Y	Z
C	-3.23500900	2.09514400	0.90023600
C	-2.37808600	0.97756100	0.42233500
C	-2.96911500	-0.27117800	0.07084100
C	-4.36919700	-0.45659800	-0.09455500
C	-5.29983100	0.62964500	-0.12317600
C	-6.65645200	0.38899900	-0.33463200
H	-7.34582600	1.22711700	-0.34297700
C	-7.12578600	-0.90034100	-0.53024500
C	-6.22824800	-1.97741100	-0.50733300
C	-4.88548800	-1.76151700	-0.29496800
H	-8.18434700	-1.07069500	-0.69062700
H	-6.59337800	-2.98817000	-0.65425000
H	-4.18862900	-2.58995100	-0.27710900
H	-2.71843900	3.05365000	0.83316600
O	-2.19485700	-1.32088700	-0.13414500
C	-1.01684500	1.12557100	0.43726200
N	-0.16200000	0.08371000	0.18534900
B	-0.76028900	-1.36831600	0.16443500
F	-0.15274900	-2.11351200	-0.83757600
F	-0.55499500	-1.96339400	1.41082800
C	3.03647800	1.83302600	-0.41496500
C	1.69003800	1.59536100	-0.29087700
C	1.18127600	0.30342800	0.04969500
C	2.08627100	-0.76992200	0.25443800
C	3.41800500	-0.49436400	0.12178200
C	3.93816800	0.77916400	-0.19923100
H	3.39212500	2.81887600	-0.69184900
H	0.99577900	2.39657000	-0.50819000
H	1.73610500	-1.75299800	0.53279200
S	-4.79148100	2.29526300	-0.01041800
H	-0.58410300	2.07641200	0.72448900
H	-3.51851100	1.93543200	1.94826200

C	5.35574700	0.62376500	-0.22022500
C	5.58644400	-0.73158900	0.09449500
C	6.44935300	1.46572900	-0.46378900
C	7.72056700	0.92620700	-0.38712900
C	7.91689900	-0.42807300	-0.07301800
C	6.84814200	-1.28439800	0.17444200
H	6.30044000	2.51181300	-0.70660200
H	8.58377700	1.55552800	-0.57190000
H	8.92722500	-0.81862300	-0.02135800
H	6.99226600	-2.32993700	0.41754900
O	4.41915200	-1.40545600	0.30265900

Table S43. The S_2 state Cartesian coordinates of S-BF2_O in CH_2Cl_2.

	X	Y	Z
C	-3.24655000	2.02432500	1.03046000
C	-2.37988400	0.92956000	0.48681200
C	-2.96723500	-0.26108800	0.03834400
C	-4.39786800	-0.44883800	-0.07544500
C	-5.29653300	0.64760800	-0.10607200
C	-6.66765500	0.47611800	-0.25790200
H	-7.31575900	1.34533700	-0.29292600
C	-7.20284900	-0.81118600	-0.37391500
C	-6.33940800	-1.89539500	-0.36590200
C	-4.95531700	-1.71777400	-0.22977900
H	-8.27084700	-0.94726900	-0.48990000
H	-6.73060100	-2.90035800	-0.48167800
H	-4.29087800	-2.57284000	-0.26193100
H	-2.72249400	2.97994400	1.06423900
O	-2.21594700	-1.30555500	-0.27485000
C	-1.01025000	1.07376900	0.49492300
N	-0.17835200	0.05752000	0.13359000
B	-0.77123300	-1.37401900	0.00461700
F	-0.17061800	-2.05259900	-1.05100000
F	-0.57145200	-2.08193500	1.20057700
C	3.03559600	1.80713800	-0.46724100
C	1.68273100	1.55705800	-0.35841500
C	1.18803500	0.28468400	0.02265500
C	2.08785900	-0.76271000	0.28913600
C	3.42705600	-0.48514200	0.15495600
C	3.93977500	0.77341900	-0.20528200

H	3.38479400	2.78828000	-0.76964000
H	0.97867000	2.34186600	-0.60796100
H	1.73660900	-1.73595800	0.60236200
S	-4.65660300	2.27238100	-0.07146300
H	-0.56126000	1.99515200	0.84867500
H	-3.65139200	1.80435700	2.02313600
C	5.36837100	0.62289100	-0.20531000
C	5.59946400	-0.71530600	0.16094500
C	6.46086100	1.45502000	-0.46597400
C	7.73647800	0.92377100	-0.35399500
C	7.93457600	-0.41423300	0.01234500
C	6.86236800	-1.26125200	0.27790600
H	6.31381000	2.49143100	-0.74947900
H	8.59829300	1.55138700	-0.55236200
H	8.94488600	-0.80057800	0.09103200
H	7.00428300	-2.29709700	0.56180900
O	4.43007800	-1.38670900	0.37911400

Table S44. The T_1 state Cartesian coordinates of S-BF2_O in CH_2Cl_2.

	X	Y	Z
C	-3.25589200	2.08174300	0.88191100
C	-2.37631000	0.98006700	0.39259300
C	-2.97066400	-0.25842000	0.02028400
C	-4.38473000	-0.46105000	-0.08114900
C	-5.30482300	0.61885700	-0.12643100
C	-6.66763700	0.37898500	-0.24826000
H	-7.35401100	1.21925600	-0.27002500
C	-7.15227200	-0.92118500	-0.35063400
C	-6.26090600	-1.99486800	-0.31736200
C	-4.90426000	-1.76946600	-0.18327600
H	-8.21830400	-1.09296200	-0.44896900
H	-6.63170300	-3.01159600	-0.39169000
H	-4.20717300	-2.59813900	-0.15669000
H	-2.73417000	3.03930000	0.87528000
O	-2.18379600	-1.30311300	-0.24958500
C	-1.01734200	1.16040700	0.40966000
N	-0.15322100	0.11694200	0.13789300
B	-0.78010100	-1.36452900	0.07931000
F	-0.13161600	-2.07699200	-0.91603700
F	-0.56180800	-1.93121200	1.33187900

C	3.05688000	1.85762000	-0.33137700
C	1.70914200	1.63382600	-0.22850600
C	1.18326700	0.32589000	0.03006600
C	2.07424400	-0.77468800	0.18682800
C	3.40924200	-0.50588800	0.09276400
C	3.94149200	0.77886300	-0.15699100
H	3.43029300	2.84986700	-0.55488700
H	1.02631100	2.45339500	-0.40621200
H	1.70875100	-1.76648400	0.40543200
S	-4.73743500	2.29158100	-0.14379500
H	-0.59632300	2.10444800	0.72490100
H	-3.57705200	1.87061600	1.90742800
C	5.36005900	0.61228400	-0.16730100
C	5.57462400	-0.75608800	0.08119100
C	6.45948100	1.45881400	-0.35466300
C	7.72600400	0.90718200	-0.28891700
C	7.90802400	-0.46243000	-0.04069600
C	6.83252200	-1.32257000	0.14951300
H	6.31965300	2.51656900	-0.54659900
H	8.59566400	1.53845800	-0.43080400
H	8.91502800	-0.86216600	0.00438300
H	6.96666000	-2.37984500	0.34154300
O	4.39983700	-1.43131600	0.24055700

Table S45. The T_2 state Cartesian coordinates of S-BF2_O in CH_2Cl_2.

	X	Y	Z
C	-3.22579300	1.98842000	1.11493100
C	-2.37427800	0.90871100	0.53462600
C	-2.97001200	-0.29544200	0.10386900
C	-4.38286100	-0.45394600	-0.07033700
C	-5.29108600	0.66699100	-0.08773000
C	-6.66198500	0.50110800	-0.35881700
H	-7.30184700	1.37736400	-0.37823300
C	-7.18557000	-0.75631000	-0.58333100
C	-6.31019600	-1.85513700	-0.57726400
C	-4.94504900	-1.70611000	-0.33946500
H	-8.24229000	-0.88943900	-0.77482900
H	-6.69884300	-2.84795100	-0.77811900
H	-4.29032300	-2.56784500	-0.37720200
H	-2.70783600	2.94758600	1.14822400

O	-2.21580700	-1.33701300	-0.20858600
C	-1.00864400	1.05057000	0.49751100
N	-0.18098600	0.03593700	0.15856800
B	-0.77032300	-1.40122400	0.07617500
F	-0.17082500	-2.11372800	-0.95493900
F	-0.57308600	-2.06503100	1.29627800
C	3.01571600	1.76420900	-0.60133900
C	1.66351800	1.50849200	-0.46528900
C	1.18923700	0.26747700	0.01501000
C	2.09689000	-0.74813000	0.34905400
C	3.43503700	-0.46794100	0.18659100
C	3.93038600	0.76370600	-0.26897600
H	3.35174700	2.72320600	-0.98026000
H	0.94894000	2.26413700	-0.77026400
H	1.75639900	-1.69925300	0.73486800
S	-4.70487100	2.27029300	0.09237100
H	-0.55448300	1.99136300	0.79307000
H	-3.59694400	1.75551900	2.11724800
C	5.36527500	0.62582000	-0.26922800
C	5.61058000	-0.67665600	0.19300100
C	6.44699900	1.44415600	-0.59872400
C	7.73005800	0.93407000	-0.45653900
C	7.94333000	-0.36950600	0.00660200
C	6.87982700	-1.20233600	0.34148500
H	6.28933700	2.45536500	-0.95799600
H	8.58386300	1.55389000	-0.70775900
H	8.95747200	-0.74065600	0.10702400
H	7.03183900	-2.21314900	0.70085600
O	4.44784900	-1.34107800	0.46967300

6.7 S-BF2_S

Table S46. The S_0 state Cartesian coordinates of S-BF2_S in CH_2Cl_2.

	X	Y	Z
C	-3.41937100	1.90768100	1.29639000
C	-2.58185700	0.84870700	0.65453500
C	-3.17544200	-0.27442100	0.09579600
C	-4.61867100	-0.38581500	-0.08256100
C	-5.45317600	0.75117900	-0.05531100
C	-6.80877300	0.61209800	-0.35070700

H	-7.45021600	1.48650400	-0.33450100
C	-7.33956200	-0.63488700	-0.64499700
C	-6.52724400	-1.76726000	-0.64689300
C	-5.17818300	-1.63716200	-0.37437400
H	-8.39874600	-0.72431300	-0.86135600
H	-6.94734800	-2.74270200	-0.86305300
H	-4.52545200	-2.50186600	-0.38533500
H	-2.84673400	2.82341500	1.45159200
O	-2.46601700	-1.26150200	-0.36677000
C	-1.19115600	0.95818900	0.59214500
N	-0.40594700	-0.01065200	0.16729200
B	-1.01198500	-1.41980200	-0.13046100
F	-0.44355100	-1.95761500	-1.26537100
F	-0.82171300	-2.24936000	0.97156500
C	2.81907400	1.64383500	-0.63640200
C	1.46119900	1.41767200	-0.51206700
C	0.98785900	0.21154500	0.02444800
C	1.87258700	-0.78667700	0.41683100
C	3.23293100	-0.54873000	0.27674500
C	3.73064100	0.66215300	-0.24100900
H	3.17059500	2.57879100	-1.05909300
H	0.75608300	2.16347800	-0.86155400
H	1.49639000	-1.71135900	0.83646400
S	-4.83179200	2.37096800	0.25321500
H	-0.71747300	1.88520800	0.90712600
H	-3.80927700	1.58086600	2.26434400
C	5.17636300	0.68053100	-0.28718200
C	5.73227200	-0.51530500	0.20344900
C	6.03273100	1.69122200	-0.73556800
C	7.40271700	1.49834400	-0.68907100
C	7.93733200	0.30246800	-0.19744900
C	7.10924500	-0.71411400	0.25263400
H	5.62474500	2.62096100	-1.11857300
H	8.06856900	2.28051300	-1.03677500
H	9.01293000	0.16581700	-0.16743200
H	7.52531200	-1.64062600	0.63237900
S	4.50982900	-1.66245700	0.71548200

Table S47. The S_1 state Cartesian coordinates of S-BF2_S in CH_2Cl_2.

	X	Y	Z

C	-3.41884900	2.10899600	0.93271400
C	-2.57779700	0.99002700	0.43228900
C	-3.18103600	-0.24148900	0.04861400
C	-4.58343800	-0.41116200	-0.11732400
C	-5.50701900	0.68192900	-0.10704400
C	-6.86709600	0.45876800	-0.31891600
H	-7.54972800	1.30213300	-0.29728600
C	-7.34691500	-0.81990700	-0.55243000
C	-6.45646900	-1.90322700	-0.56850000
C	-5.11068900	-1.70445100	-0.35635400
H	-8.40737400	-0.97783400	-0.71253400
H	-6.82961000	-2.90614500	-0.74584700
H	-4.42038400	-2.53837700	-0.36873200
H	-2.89458200	3.06374500	0.87110000
O	-2.41694900	-1.29240800	-0.18977300
C	-1.21293800	1.12112000	0.45904600
N	-0.37045300	0.07756700	0.18914400
B	-0.98436900	-1.36590400	0.10858700
F	-0.37969400	-2.07608500	-0.92041000
F	-0.78798300	-2.01336500	1.33081000
C	2.86394400	1.76878800	-0.34610000
C	1.51138400	1.56970500	-0.23854100
C	0.97785300	0.28524100	0.06763000
C	1.87006200	-0.79911400	0.25589600
C	3.22202700	-0.57481400	0.14449800
C	3.75964400	0.70156700	-0.15148700
H	3.24102200	2.75366600	-0.59961300
H	0.84091500	2.39455900	-0.44267200
H	1.48161400	-1.77380900	0.51573300
S	-4.98844700	2.33819600	0.05504600
H	-0.77085300	2.06173300	0.76546200
H	-3.68851900	1.93979400	1.98347000
C	5.18803300	0.69054000	-0.21033000
C	5.71859900	-0.59575500	0.05156700
C	6.07238200	1.74802000	-0.48142900
C	7.43242800	1.51364900	-0.48716500
C	7.93797100	0.23174300	-0.22431100
C	7.08838400	-0.83098200	0.04619400
H	5.68575300	2.74056900	-0.68612200
H	8.11911400	2.32608000	-0.69676400

H	9.00976400	0.06628900	-0.23260700
H	7.48536200	-1.81934900	0.24787000
S	4.47434700	-1.78428100	0.36277000

Table S48. The S_2 state Cartesian coordinates of S-BF2_S in CH_2Cl_2.

	X	Y	Z
C	-3.44079400	2.02430700	1.08254500
C	-2.58210900	0.93551200	0.51414600
C	-3.18027800	-0.23591400	0.02984200
C	-4.61175400	-0.40346000	-0.09903100
C	-5.49682200	0.70409300	-0.10713400
C	-6.86832800	0.55240100	-0.27370900
H	-7.50632200	1.42962400	-0.28992600
C	-7.41722300	-0.72493700	-0.42892700
C	-6.56689500	-1.81958300	-0.44287000
C	-5.18263200	-1.66184600	-0.29098400
H	-8.48580000	-0.84507000	-0.55702300
H	-6.96912700	-2.81635600	-0.58798700
H	-4.52754600	-2.52335200	-0.33961800
H	-2.90548700	2.97206500	1.14816300
O	-2.43864000	-1.28043400	-0.30618900
C	-1.21134200	1.06348100	0.53495800
N	-0.38835100	0.04806100	0.15074400
B	-0.99605100	-1.37182600	-0.02722300
F	-0.39857500	-2.02075700	-1.10384400
F	-0.80493200	-2.12239200	1.14441900
C	2.85824000	1.74765900	-0.37511100
C	1.49902600	1.53422100	-0.28181100
C	0.97990000	0.26391100	0.05055000
C	1.87064100	-0.80053900	0.28031100
C	3.22785800	-0.57018200	0.16460100
C	3.75757800	0.69635600	-0.15290400
H	3.22762600	2.73266500	-0.64103300
H	0.81838300	2.34697200	-0.50580500
H	1.48448800	-1.77125200	0.56141900
S	-4.83756300	2.32052200	-0.02466800
H	-0.75368400	1.96962200	0.91600900
H	-3.85558200	1.78026400	2.06533900
C	5.19572800	0.69234400	-0.19997200
C	5.72976400	-0.58351600	0.09167400

C	6.07727300	1.74429800	-0.48204900
C	7.44067700	1.51601300	-0.47038400
C	7.95127200	0.24336200	-0.17818700
C	7.10225800	-0.81475800	0.10487000
H	5.68988300	2.73198200	-0.70901400
H	8.12440100	2.32884800	-0.68928800
H	9.02383500	0.08260100	-0.17342700
H	7.49860900	-1.79849200	0.33020700
S	4.48653100	-1.76958000	0.41308600

Table S49. The T_1 state Cartesian coordinates of S-BF2_S in CH_2Cl_2.

	X	Y	Z
C	-3.44328100	2.09403200	0.91978300
C	-2.57516100	0.99182600	0.41251900
C	-3.18265700	-0.23155800	0.01131300
C	-4.59831600	-0.41503100	-0.09957600
C	-5.50564400	0.67650200	-0.12548100
C	-6.87064800	0.45523400	-0.25776400
H	-7.54710900	1.30374900	-0.26445200
C	-7.36987700	-0.83667600	-0.38959700
C	-6.49116600	-1.92137500	-0.37566900
C	-5.13268600	-1.71494800	-0.23154600
H	-8.43740400	-0.99384700	-0.49571400
H	-6.87358700	-2.93182600	-0.47299200
H	-4.44546200	-2.55214700	-0.21979300
H	-2.91004600	3.04510900	0.93429900
O	-2.40715200	-1.27927100	-0.27770500
C	-1.21358300	1.15488600	0.44023700
N	-0.36172200	0.10779700	0.15205200
B	-1.00429500	-1.36415600	0.04928500
F	-0.36104300	-2.05309600	-0.96576900
F	-0.79352400	-1.97061300	1.28466700
C	2.87834000	1.79182800	-0.26371500
C	1.52697500	1.60568500	-0.17027900
C	0.97858800	0.30496100	0.05480300
C	1.85828800	-0.80384400	0.19090800
C	3.21338900	-0.58639100	0.11009500
C	3.75995400	0.70084700	-0.11590600
H	3.27058900	2.78243000	-0.46398300
H	0.86641100	2.44717000	-0.32937400

H	1.45731700	-1.78650200	0.39142200
S	-4.91902000	2.34229200	-0.10555500
H	-0.78262300	2.08855600	0.77266900
H	-3.77026900	1.86611200	1.93994100
C	5.19017300	0.68178100	-0.16269500
C	5.70637800	-0.61822400	0.03829400
C	6.08062000	1.74623000	-0.37299800
C	7.43939700	1.50330700	-0.37871800
C	7.93255100	0.20645200	-0.17620800
C	7.07527700	-0.86311200	0.03285300
H	5.70185600	2.75004600	-0.53137700
H	8.13326000	2.32013900	-0.54132700
H	9.00322900	0.03409500	-0.18345600
H	7.46432000	-1.86277300	0.18789100
S	4.45213900	-1.81298100	0.27887300

Table S50. The T_2 state Cartesian coordinates of S-BF2_S in CH_2Cl_2.

	X	Y	Z
C	-3.41725400	1.97724000	1.17639500
C	-2.57546800	0.90833700	0.56253600
C	-3.18355400	-0.27255000	0.08645900
C	-4.59727200	-0.40796900	-0.09976800
C	-5.49168600	0.72358900	-0.08179100
C	-6.86305400	0.58419200	-0.36469500
H	-7.49237700	1.46816000	-0.35569700
C	-7.40058800	-0.65814500	-0.63611100
C	-6.53852300	-1.76678400	-0.66567900
C	-5.17278200	-1.64281300	-0.41643800
H	-8.45789600	-0.77147400	-0.83686900
H	-6.93784100	-2.74699400	-0.90400700
H	-4.52811500	-2.51042200	-0.48244100
H	-2.88812200	2.92839900	1.24581800
O	-2.44041800	-1.31132500	-0.25938100
C	-1.20805100	1.03425800	0.53831800
N	-0.39055300	0.02228100	0.16818800
B	-0.99683900	-1.40316100	0.02589100
F	-0.40214200	-2.08057200	-1.03131000
F	-0.81121800	-2.11805200	1.21890600
C	2.84161300	1.71434400	-0.48696600
C	1.48450100	1.49186700	-0.37428600

C	0.98349400	0.24100500	0.04383400
C	1.87811700	-0.79577500	0.33263100
C	3.23644400	-0.55985000	0.19856100
C	3.75036600	0.68957000	-0.20047900
H	3.19944400	2.68341500	-0.81897200
H	0.79272300	2.28021900	-0.64766000
H	1.50234000	-1.75175200	0.67306500
S	-4.88646300	2.31232500	0.15565300
H	-0.74475000	1.95832500	0.87017500
H	-3.79697700	1.71370000	2.16780400
C	5.19282100	0.69631400	-0.25614400
C	5.74002400	-0.54948800	0.10981400
C	6.06036800	1.73663800	-0.60847700
C	7.42845100	1.52493500	-0.59197100
C	7.95279500	0.28059200	-0.22557300
C	7.11400500	-0.76646300	0.12826100
H	5.66091000	2.70445700	-0.89383500
H	8.10099300	2.33067900	-0.86544500
H	9.02684500	0.12942200	-0.21742800
H	7.52164800	-1.73062200	0.41147400
S	4.50610100	-1.72768200	0.51455600

References

(1) Hehre, W. J.; Ditchfield, R.; Pople, J. A. Self-consistent molecular orbital methods. XII. Further extensions of Gaussian-type basis sets for use in molecular orbital studies of organic molecules. *J. Chem. Phys.* **1972**, *56*, 2257–2261.

(2) Hariharan, P. C.; Pople, J. A. The influence of polarization functions on molecular orbital hydrogenation energies. *Theor. Chimi. Acta.* **1973**, *28*, 213–222.

(3) Sun, J.; Ruzsinszky, A.; Perdew, J. Strongly Constrained and Appropriately Normed Semilocal Density Functional. *Phys. Rev. Lett.* **2015**, *115*, 036402.

(4) Tozer, D. J.; Peach, M. J. G. Molecular excited states from the SCAN functional. *Mol. Phys.* **2018**, *116*, 1504–1511.

(5) Brédas, J.-L.; Beljonne, D.; Coropceanu, V.; Cornil, J. Charge-transfer and energy-transfer processes in π-conjugated oligomers and polymers: a molecular picture. *Chem. Rev.* **2004**, *104*, 4971–5004.

(6) Samanta, P. K.; Kim, D.; Coropceanu, V.; Bredas, J.-L. Up-conversion intersystem crossing rates in organic emitters for thermally activated delayed fluorescence: impact of the nature of singlet vs triplet excited states. *J. Am. Chem. Soc.* **2017**, *139*, 4042–4051.

(7) Abroshan, H.; Coropceanu, V.; Brédas, J.-L. Radiative and Nonradiative Recombinations in Organic Radical Emitters: The Effect of Guest–Host Interactions. *Adv. Func. Mater.* **2020**, *30*, 2002916.

(8) Veys, K.; Escudero, D. Computational Protocol To Predict Anti-Kasha Emissions: The Case of Azulene Derivatives. *J. Phys. Chem. A* **2020**, *124*, 7228–7237.

(9) Cave, R. J.; Newton, M. D. Generalization of the Mulliken-Hush Treatment for the Calculation of Electron Transfer Matrix Elements. *Chem. phys. lett.* **1996**, *249*, 15–19.

(10) Rust, M.; Lappe, J.; Cave, R. J. Multistate Effects in Calculations of the Electronic Coupling Element for Electron Transfer Using the Generalized Mulliken- Hush method. *J. Phys. Chem. A* **2002**, *106*, 3930–3940.

(11) Lu, T.; Chen, F. Multiwfn: A multifunctional wavefunction analyzer. *J. Comput. Chem.* **2012**, *33*, 580–592.

(12) Peng, Q.; Fan, D.; Duan, R.; Yi, Y.; Niu, Y.; Wang, D.; Shuai, Z. Theoretical study of conversion and decay processes of excited triplet and singlet states in a thermally activated delayed fluorescence molecule. *J. Phys. Chem. C* **2017**, *121*, 13448–13456.
